# Measles and the canonical path to elimination

**DOI:** 10.1126/science.aau6299

**Published:** 2019-05-10

**Authors:** Matthew Graham, Amy K. Winter, Matthew Ferrari, Bryan Grenfell, William J. Moss, Andrew S. Azman, C. Jessica E. Metcalf, Justin Lessler

**Affiliations:** 1 Department of Epidemiology, Johns Hopkins Bloomberg School of Public Health, Baltimore, MD, USA; 2 Department of Biology, The Pennsylvania State University, University Park, PA, USA; 3 Department of Ecology and Evolutionary Biology, Princeton University, Princeton, NJ, USA; 4 Fogarty International Center, National Institutes of Health, Bethesda, MD, USA

## Abstract

All World Health Organization regions have set measles elimination goals. We find that as countries progress toward these goals, they undergo predictable changes in the size and frequency of measles outbreaks. A country’s position on this “canonical path” is driven by both measles control activities and demographic factors, which combine to change the effective size of the measles-susceptible population, thereby driving the country through theoretically established dynamic regimes. Further, position on the path to elimination provides critical information for guiding vaccination efforts, such as the age profile of susceptibility, that could only otherwise be obtained through costly field studies or sophisticated analysis. Equipped with this information, countries can gain insight into their current and future measles epidemiology and select appropriate strategies to more quickly achieve elimination goals.

Building on initially successful efforts to eliminate measles in the Americas, as of 2013, all World Health Organization (WHO) regions established measles elimination goals (*1*). In doing so, the global health community has effectively set a goal of measles eradication by 2020 (*1*). However, elimination has never been achieved in five of the six WHO regions and a recent resurgence of measles in Venezuela (>5000 cases in 2018) and Brazil (>2000 cases in 2018), which has led to the loss of elimination status in the Americas, raises concerns about the sustainability of elimination (*2*). As progress is made toward elimination, knowledge of local measles virus transmission dynamics can inform critical decisions in measles control policy, such as at what age to administer a second dose of measles-containing vaccine (MCV) and what ages to target during vaccination campaigns (*3*, *4*). Here, we characterize the dynamics of measles incidence between 1980 and 2017 in all countries as they move along the path toward measles elimination, highlighting commonalities across countries and implications for measles control policy.

When tracking progress toward measles elimination, incidence is typically the primary, if not only, method by which each country’s changing disease dynamics is characterized. However, this approach fails to capture essential aspects of the dynamical system, notably changing multiannual patterns of incidence (*5*, *6*). In some cases, complex dynamic models are fit to data from individual countries to give an in-depth and nuanced picture of their dynamics (*7*–*9*), but such efforts are labor intensive, technically demanding, and require detailed, fine-scale data that are often not readily available. We take an intermediate approach, characterizing each country’s dynamic regime by its position in a two-dimensional (x,y) space defined by the mean and inter annual coefficient of variation (CV) of its measles incidence. We refer to this as the “incidence space.”

To determine a country’s position in incidence space in a given year, we calculate each country’s y position as the average of its WHO-reported incidence every year since 1980 weighted by a truncated Gaussian distribution peaking 2 years prior (figs. S1 and S2) (*10*). For the x position, at each year, we calculate its year-to-year CV over the previous 10 years and then weight these by the same Gaussian distribution (figs. S1 and S2) (*10*). Thus, we capture the non stationarity in measles dynamics for each country and reflect the gradual transition between dynamic regimes Fig. 1). This approach allows us to analyze global patterns of measles dynamics between 1990 and 2017 and uncover similarities between countries, using readily available data (if of differing quality) for every country in the world (*11*). Note that 1990 is the earliest date for which we calculate positions in incidence space, because each point is supported by the prior 10 years of data.

**Fig. 1:**
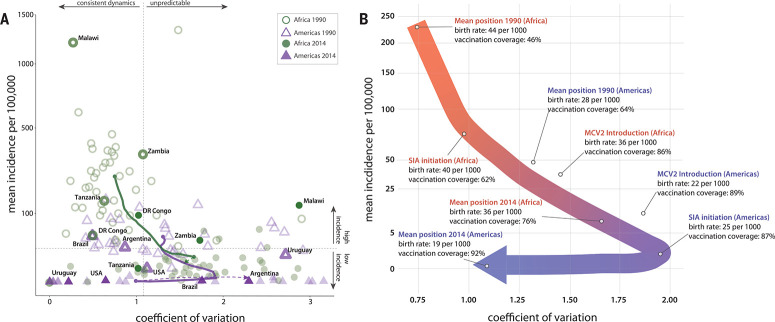
**Characterizing the canonical path to elimination.** (**A**) Location of countries in the WHO African Region and Region of the Americas in “incidence space” in 1990 and 2014. As a country’s position on the x axis moves to the right, the unpredictability of the year-to-year cases increases. Arrows show the mean trajectory of Africa and Americas Region countries in green and purple, respectively, for each year in the intervening period, with dotted lines showing movement after 2014. (**B**) Combining the mean paths of Africa and the Americas, an almost continuous path toward elimination is observed, shown by the thick arrow moving from red to blue. Along this path, vaccination coverage and birth rate are labeled, corresponding with the initial position in 1990, the final position in 2014, and the introduction of both supplemental immunization activities and a second routine dose of measles-containing vaccine for both the Americas and Africa.

Using age-stratified compartmental models to simulate expected annual incidence based solely on changing demography (birth and death rates, size and age structure) and reported vaccination coverage, we show that countries are expected to follow a “canonical path” through this space (figs. S3 to S12). Countries begin in a regime of uncontrolled endemic transmission, characterized by high incidence and low year to- year variability, then progress through regions of lower incidence but high year-to-year variability, finally settling near the point of elimination (zero incidence, zero variation). Empirically observed patterns broadly match these simulations (Fig. 1), with observations having higher variability than simulated and, in smaller countries, lower incidence than expected (figs. S3 to S12 directly compare paths). Deviations between simulated and observed pathways may be the result of processes not captured in the model (e.g., the interdependence of measles dynamics between countries in the same region), simplifying assumptions (e.g., homogeneous geographic mixing within countries), or uncertainty in the underlying data (particularly vaccination coverage), as well as the inherent variability in the disease process. Many of these processes, particularly influence from surrounding countries, are expected to be more pronounced for smaller countries. Despite deviations between observed and simulated patterns, these observations are robust to using median instead of mean to report the average position- and country-specific corrections for underreporting of measles cases using a state-space model (*12*, *13*) (figs. S13 and S14).

Africa and the Americas exemplify opposite ends of the canonical path toward measles elimination, and we focus on these regions to define and explore this path (Fig. 1A). The Americas precede Africa on the path to elimination; the location of the Americas on the path in 1995 coincides almost exactly with that of Africa in 2008. Therefore, to concretely define the points on the canonical path, the progression of the yearly mean position of all African countries from 1990 to 2007 was joined with that of all American countries from 1995 to 2014 (so as to exclude the 2015–2017 measles resurgence in the Americas when defining the path) (Fig.1A). Figure 1B displays a schematic of these two regional pathways combined to create the derived empirical pathway.

Once we define the specific canonical pathway in incidence space, we can analyze the progress of a country, or region, along this path. To approximate a country’s position on the (one dimensional) canonical path at any given time, we project its position in (two-dimensional) incidence space that year onto the nearest (i.e., closest in incidence space) point on the canonical path (Fig. 2 and figs. S15 and S16). Each position along the path can then be characterized by the percentage of the path completed, with the average position of African countries in 1990 representing 0%, and the position of the Americas in 2014 (before the 2015 measles resurgence) representing 100% (Fig. 3A and fig. S17A). Likewise, we can use these projections to capture each country’s speed of progression along the path (Fig. 3B and fig. S17B). These trajectories capture global trends in measles epidemiology, with all WHO regions progressing steadily along the path (Fig. 3, A and B), and particularly rapid progress toward elimination in the Americas during the 1990s and Africa in the mid- to late 2000s. This movement has caused major shifts in the measles dynamics experienced by the world’s population between 1990 and 2017, from nearly everyone living in high-incidence, low variance countries to most living in countries with low incidence, although relatively few have reached stable elimination (Fig. 2). However, there are exceptions to this forward progress, most notably backward movements in the Americas associated with outbreaks between 2015 and 2017.

**Fig. 2:**
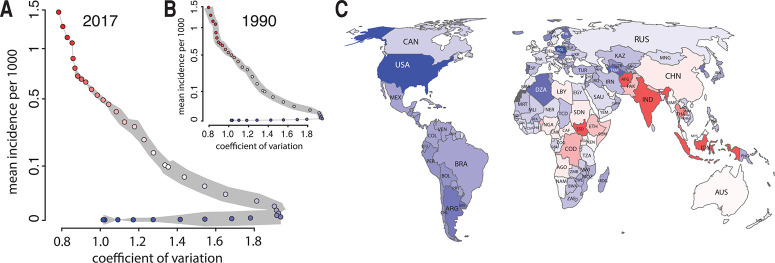
**Progression along the path of the world’s population.** (**A** and **B**) The position along the canonical path is indicated by the corresponding color as shown in (A).The thickness of the gray shading around the path is proportional to the log-population residing in countries reaching that point on the path by 2017 (A) and 1990 (B). (**C**) Each country’s position along the path as of 2017.

**Fig. 3:**
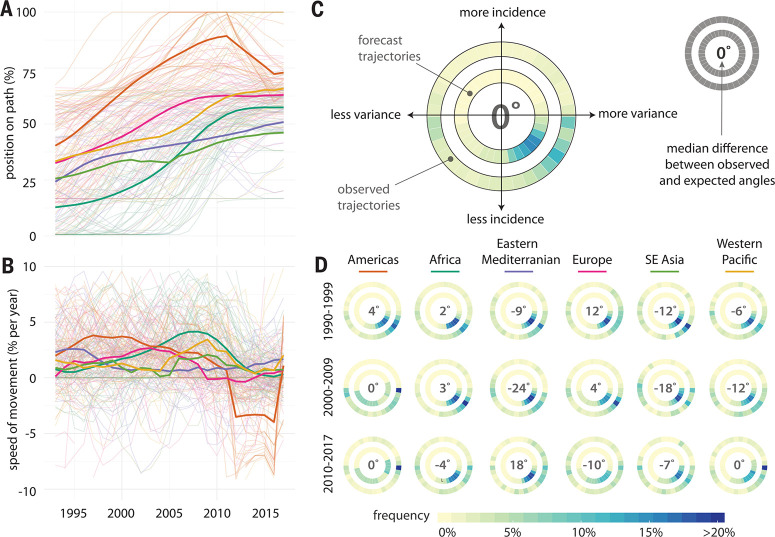
**Analysis of movement along the canonical path.** (**A**) The position of each country (light lines) and mean position of each WHO region (heavy lines) along the canonical path, measured as percentage complete. (**B**) The speed of movement along the path as percentage per year. (**C**) Heat map of the predicted angle of movement in incidence space based on the projected position of each country onto the canonical path in each year (inner circle) versus the actual observed angles of movement (outer circle). Bluer shades represent a higher proportion of countries moving (or being predicted to move) in that direction. The inner number shows the median difference between the predicted and observed angles of movement across all countries and years. (**D**) Predicted and observed angles of movement among all countries in each WHO region in each decade period since 1990.

On the basis of their position along the path, we can also determine the direction in which each country is expected to move in incidence space if they follow the canonical path (for countries at the end of the path, movements are considered to be consistent with expectation if they maintain a lower CV and incidence than the final position). Predictions of the direction of movement are unbiased; over the entire period of analysis, the median deviation of countries from their expected trajectory was 0° [interquartile range (IQR) –57, 31] (Fig. 3C). Path position predicted movement in the same general direction (±45%) as actual movement most of the time (54%, figs. S18 and S19, table S1, and supplementary text). Decade-specific regional variations were sometimes more pronounced (Fig. 3D), with the highest deviations being in the WHO Eastern Mediterranean Region between 2000 and 2009 (–24°, IQR –119, 10). There were also notable unexpected movements in the Americas between 2010 and 2017 associated with measles resurgence in the region; 25% of countries had a deviation of >78°, although the median deviation was still 0°. A country’s position in incidence space relative to its projected position on the path was unbiased in both incidence and CV dimensions, and on average, a country’s distance from the path was <10% of the path length (figs. S20 and S21 and supplementary text).

Increasing vaccination coverage and decreasing birth rates both play an important role in driving countries toward measles elimination, as both decrease the rate of replenishment of susceptible individuals. Both are highly predictive of a country’s position in incidence space, and thus its progress on the canonical path (figs. S22 and S23). The impact of susceptibility also underpins a fundamental relationship between community size and measles dynamics (*5*, *14*) identified by Bartlett (*15*) and is characterized as follows: Large populations have type I dynamics (regular epidemics), midsized communities have type II dynamics (occasional fadeouts in transmission), and small communities have type III dynamics (sporadic epidemics) (*16*). The classification of a large, medium, or small community depends on the disease and local demographics. In unvaccinated populations, the “critical community size” for measles, above which type I dynamics can be sustained (i.e., measles can continue to circulate without reintroductions), has been estimated to be between 250,000 and 500,000 in most settings (*15*, *17*), with a higher threshold in cases of highly seasonal transmission (*7*). Vaccination decreases the effective size of the community through elimination of susceptible individuals, thereby increasing the critical community size (*18*, *19*), whereas higher birth rates decrease the critical community size by more quickly replenishing the susceptible population. The canonical path mirrors this classic taxonomy, with countries starting at the upper left corner in incidence space (Fig. 1), with regular epidemics (type I dynamics), then showing decreasing incidence and increasing variation (type II then III dynamics) as vaccination and decreasing birth rates drive up the critical community size, and finally settling at a low variance and low-incidence regime as elimination is achieved (i.e., long-term absence of endemic measles transmission) (*5*, *14*, *15*).

A country’s position on the canonical path is not solely of theoretical interest but can also inform specific disease control policies, helping these countries to keep on the path to elimination and potentially accelerating progress. For example, this position is linked to the likely age distribution of individuals susceptible to measles in a country, a key piece of information for designing mass vaccination campaigns (known as supplementary immunization activities, or SIAs). Typically, the age range of SIAs is chosen on the basis of the age distribution of confirmed cases in previous years (*4*). However, this approach dooms us to always be “fighting the last war,” as illustrated by outbreaks in Angola (2009) and a large outbreak in Malawi (2010), where the age range of cases was far wider than in previous years (fig. S24). Analysis of a country’s position along the path to elimination provides an alternative approach to estimating the underlying age distribution of immunity (which could be supplemented and validated with serological data).

To estimate the susceptible population, we link data on age-specific measles incidence [corrected for underreporting (*12*, *13*)], yearly birth rates, and vaccination [both routine and supplementary (*20*)]. By appropriately scaling incidence and variation (*10*) and projecting countries onto the closest point on the canonical path (Fig. 2), the distribution of susceptible individuals typical of countries at each position on the path can be estimated with a state-space model (*13*) (Fig. 4). Comparisons of these susceptibility profiles with the age distribution of cases in outbreaks, where available, show good agreement (Fig. 4B).

**Fig. 4:**
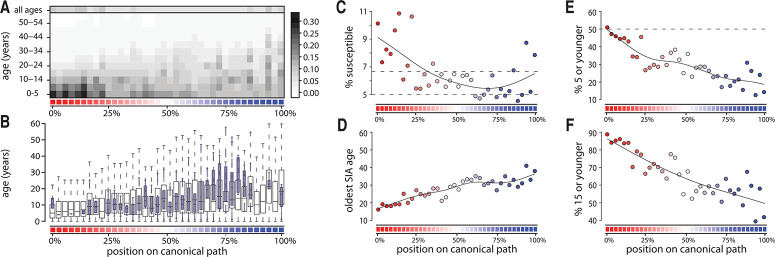
**Progression along the path and the age of susceptibility.** In all plots, the position along the canonical path is indicated by the corresponding color as shown in Fig. 2A and percentage complete. (**A**) Age-specific proportion susceptible for each position on the canonical path.The legend on the right side of the figure, correlated with the colors in the plot, represents the estimated age-specific proportion susceptible. (**B**) The mean age of susceptibility to measles estimated by our approach (hollow wide boxes) and the mean age of cases for countries reporting these data around the world from 1995 to 2016 (filled narrow boxes) by canonical path position. (**C**) Proportion of the population susceptible, with dashed horizontal lines representing the level of susceptibility above which herd immunity is compromised when assuming basic reproduction numbers of 15 and 20. (**D**) The oldest age that would need to be targeted by an SIA to cover 90% of the susceptible population. (**E**) The proportion of susceptible individuals 5 years of age or younger and (**F**) the proportion age 15 or younger.

As countries progress along the canonical path, the estimated proportion of the population susceptible decreases, but the average age of susceptible individuals increases, reflecting increasing measles susceptibility in older age cohorts (Fig. 4). Therefore, the ages that an SIA would have to target to cover 90% of those susceptible individuals increases (Fig. 4D). At almost all positions along the canonical path, the median age of susceptibility is at least 5 years. Therefore, SIAs targeting individuals up to 5 years of age likely miss more than half of susceptible individuals (Fig. 4E; Fig. 4F shows a similar analysis for those under 15 years of age).

Introduction of a second routine dose of MCV (MCV2) and SIAs targeting all children in a particular age group are also key strategies for accelerated movement down the canonical path to elimination. SIAs were first introduced in African countries during times of relatively low routine measles vaccination coverage and high birth rates (62% and 40 per 1000, respectively, averaged over the African continent, Fig. 1B), whereas in the Americas, SIAs were introduced when there was already high routine measles vaccination coverage and low birth rates (87% and 25 per 1000, respectively, averaged over the Americas). These differences in routine coverage and birth rates may partly explain why SIA-driven strategies have not always had the same sustained impact in Africa that they have had in the Americas. Introduction of MCV2 has not been fully distributed across Africa, and in the countries where it has been introduced, this was done so during times of similar routine vaccination but higher birth rate than in the Americas (86% and 36 per 1000 for Africa versus 89% and 22 per 1000 for the Americas, respectively). It is likely that these differences in part drive the observation that countries in Africa that introduce MCV2 fail to reach positions along the path comparable to those in the Americas within 10 years of introduction (fig. S25), as well as, in some cases, slow movement.

As a public health tool, position in incidence space and corresponding progress on the canonical path can provide general guidance to countries as to likely underlying susceptibility profiles and future patterns of incidence based on the experience of other countries that are, or have been, in a similar situation. By using this information, countries may be able to optimize programs and accelerate their progress toward measles elimination. Both path progress and position in incidence space could serve as important features for classifying or taxonomizing countries on the basis of their measles situation and progress toward elimination. Further, by linking these categories to our theoretical understanding of disease dynamics, thereby clarifying many drivers of observed dynamics, this work should help to better inform rational measles control strategies.

Limitations of this work include inconsistencies in measles reporting between countries and over time. However, with few exceptions, corrections for underreporting yield qualitatively identical results (figs. S26 to S29). Assignment of a position and speed of movement on the canonical path is sensitive to the scaling of incidence and CV (fig. S29) (*10*). Although alternative scalings do not change the major conclusions of this work, rescalings (particularly of incidence) do change the relative influence of incidence and variation in path position, qualitatively changing the position of some countries and regions. The canonical path captures national-level phenomena, and is not inconsistent with subnational chaotic or high-variance behavior in high-incidence settings [e.g., (*7*, *21*)], driven by local demographics and epidemiology. Further, the path represents a general pattern of changing dynamics as countries progress toward elimination; considerable deviations from the path are possible, particularly when there are changes in the drivers of elimination (e.g., declining vaccination) or substantial measles epidemics in neighboring areas.

This work shows how an assessment of a country’s measles dynamics based solely on measuring average and year-to-year variation in incidence can yield deep insights into that country’s progress toward elimination and future epidemic dynamics. Custom, in-depth analyses have an ongoing and essential role to play in strategic planning for measles elimination and our understanding of health systems in general. However, a broader view reveals important aspects of how, over time, countries move toward and achieve (or fail to achieve) their health goals [see Hans Rosling’s work on poverty and health (*22*)]. This analysis emphasizes that birth rate, in addition to vaccination, likely plays an essential role in each country’s progress toward measles elimination, although these parameters are closely associated with other factors such as economic development. Theory tells us that we should expect changes in epidemic dynamics as countries approach elimination, including more erratic epidemics and a shifting age range of infections. Only by taking a broad look at all countries over several decades do we see the dynamic regime that each country is currently experiencing and the timing and pace at which they are likely to flow through these regimes. With this knowledge, each country can better tailor interventions to its particular situation, thereby accelerating progress along the pathway to measles elimination.
