# Supplementary material for: Measles and the canonical path to elimination
Source: Science. 2019 May 10;364(6440):584–7. doi: 10.1126/science.aau6299 (PMC7745123; doi:10.1126/science.aau6299)
Supplement: Supplementary file 1 [file SCIENCE-364-6440-584-s001.pdf]

Science AAAS

science.sciencemag.org/content/364/6440/584/suppl/DC1

Supplementary Materials for

Measles and the canonical path to elimination

Matthew Graham, Amy K. Winter, Matthew Ferrari, Bryan Grenfell, William J. Moss, Andrew S. Azman, C. Jessica E. Metcalf, Justin Lessler*

*Corresponding author. Email: justin@jhu.edu

Published 10 May 2019, *Science* **364**, 584 (2019)
DOI: 10.1126/science.aau6299

This PDF file includes:

Materials and Methods
Supplementary Text
Figs. S1 to S29
Table S1
References

Materials and Methods

Data

We used data on population, birth rates, vaccination rates and measles cases by country and year from 1980. Population and birth rate data by country and year were available from the World Bank (*23*, *24*). Annual measles vaccination and case data by country were available from the WHO (*25*, *26*).

Calculation of mean incidence and coefficient of variation of incidence for incidence-space

To calculate the mean incidence over a period of years, we did not calculate a moving average of the incidence in the given period, shifting by 1 year each time. This would result in fast, non-smooth shifts in values of mean incidence as years come into and drop out of our time window after a period of time. Instead we took a weighted mean which never fully discounts a year in the past, though diminishes its impact considerably over time. We began by calculating the incidence of cases per 100,000 people for each year, which is given by the number of cases in the year divided by the population size for that year (Fig. S1A shows this for Nigeria). We wished to calculate the weighted mean incidence over the previous y years, and analysis began when we had at least y years of data. For our analysis, we set $y = 10$, so the first year we included in the analysis was 1990 for which we calculated the weighted mean incidence for years 1981-1990. To calculate the mean incidence over the previous y years, we multiplied this time series by a truncated Gaussian distribution with a standard deviation of 3 years peaking 2 years before the year in question (Fig. S1B shows examples of this Gaussian). The result of this example is shown in Fig. S1C.

The coefficient of variation of the incidence (CV) is defined as the ratio of the standard deviation of incidence by the mean of incidence. To calculate the weighted CV, we used the same Gaussian functions to estimate the weighted standard deviation and weighted mean incidence. However, when using the described Gaussian distributions, if we see an extended period of time where cases are far lower than had previously been seen (such as when approaching elimination), will result in a sharp increase in the weighted CV. This does not occur using unweighted CV when there is an unbroken chain of zeros for the entire length of the given period (i.e., 10 years) (see Fig. S2A for a comparison of these two methods for Bolivia as it approached elimination).

To avoid this problem, we instead began by calculating the unweighted CV for each 10-year period (e.g. for the 1990 entry we calculated the CV of cases for years 1981-1990). We call these the “local CV” for each year (see Fig. S2B for example from Nigeria). These local CV values were then weighted using similar Gaussian distributions to those previously described; however, the Gaussians differ in that we did not wait until we had 10 years’ worth of previous points to use (see Fig. S2C for example from Nigeria).

To generate the canonical path (see Fig. 1B & 2A for examples), this process was applied to all countries in Africa and the Americas. Following that the mean point in incidence space for the countries in both of these continents was taken each year (see green and purple arrows in Fig. 1A for the mean position of Africa and the Americas over time respectively, and Fig. S13 for the median positions). To generate the canonical path, these arrows are joined at the point at which they cross, giving a picture of what progression from endemicity of measles (as seen in Africa at the beginning of the data) to near elimination (seen in the Americas at the end of the data) would ideally look like. This linked path is what is seen in Fig. 1B. In Fig. 2A, each yearly point on the path is shown, with an adjustment in the mean incidence so that the individual incidences lie in the same range as the coefficient of variation.

<u>Scaling of incidence and CV for placing countries on the canonical path</u>

Assigning countries to the nearest point on the canonical path first required that the incidence and CV are on the same scale. At non-scaled values, incidence will dominate each country's point on the path due to the larger range in incidence compared to the CV. Additionally, for countries with low incidence it is difficult to differentiate between their points on the path. To remedy this, we took the natural log of the incidence values, which gives us much more resolution for countries with low levels of incidence, and scaled both the logged incidence and the CV so that all of their values are between 0 and 1 (Fig. S15). Figure S16 depicts the same results as Fig. 1, although includes this scaling, with an added dashed line to show the mean position of the Americas from 2015-2017, which regresses with respect to the canonical path, due to an increased number of cases in these years. This scaling was used for the construction of Fig. 2C, Fig. 3, and Fig. 4 in the main text.

We conducted a sensitivity analysis of two additional scaling techniques: i) taking the square root of incidence values and then scaling both square rooted incidence and coefficient of variation so that all of their values are between 0 and 1, and ii) taking the natural log of incidence values and square root of the coefficient of variation and again scaling both so that all of their values are between 0 and 1. The results of the sensitivity analysis, displayed via position and speed of movement by country over time (methods discussed below), are shown in Fig. S29.

<u>Calculating a granular version of the canonical path</u>

Along with the canonical path with a point per year (38 points), with distances between points being allowed to vary depending on the data, we also created a version of the path which added four equally spaced points between the original path points (186 granular points). This was done in order to be able to more accurately discuss the speed of movement along the path, and the expected versus observed movement of countries along the path. Without this additional path, a country moving by a certain amount near one part of the path could transition through a different number of points in the path compared with a country moving the exact same amount in another part of the path. This granular path can be seen in Fig. S15 as the black dotted line.

Simulation of country-specific measles incidence

We used an established discrete time age-structured mathematical model, introduced in (*27*, *28*) to simulate measles transmission dynamics for each country in the WHO Americas and Africa Regions. The key feature of the model is a matrix that at every time-step defines transition from every possible epidemiological stage (e.g. maternally immune, susceptible, infected, recovered, and vaccinated) and age combination to every other possible epidemiological stage and age combination. The matrix captures transitions between each epidemiological stage within each age class and discrete time-step, where the time-step was set to the approximate generation time of measles, about 2 weeks, assuming 24 infection generations in a year.

Simulations were based on country-specific reported data, including demographic (population age distribution, birth rate, death rate) (*29*) and vaccination coverage data (*25*), and shared empirically derived measles epidemiologic parameters, including rate of decline in maternal immunity (*30*), vaccination efficacy (*31*), basic reproductive number (R_0=12), and age-contact rates (*32*), but were otherwise completely model driven. Starting conditions were based on the distribution of immunity resulting from simulations of 30 years of uncontrolled measles in a population with a constant birth rate and an age distribution equivalent to that in the country as of 1980.

The model outputs the number of individuals in each age class and epidemiological stage at every time-step, allowing us to directly extract the number of measles cases per age and time-step. Cases were aggregated over each year and divided by the mid-year population to estimate measles incidence per year and country. Each country was stochastically simulated 100 times. We then calculate the weighted mean incidence and weighted coefficient of variation of incidence as described above using a Gaussian distribution. Figure S3-S12 displays the result of this simulation and compares it to the incidence space based on estimated country incidence over time correcting for under-reporting using a state space model (*12*, *13*), see below for more information.

Estimating measles attack rates and population susceptibility

As we wished to link the position in incidence-space to a distribution of susceptibility, we first needed country-specific susceptibility profiles. The susceptibility in the population is essentially unobservable, but we can use reported cases to infer the susceptibility over time. Simons *et al.,* 2012 fit a dynamic transmission model to correct for under-reporting of measles cases, giving an estimate of true measles incidence rather than reported incidence. An extended Kalman filter was then used to infer what the number of susceptible individuals is over time (*13*). To generate the profile of susceptibility, it is assumed that the probability of natural infection by age is given by P(infection by age a) = $1 - exp(-0.149 \times a)$. Combining this with information on birth rates, supplementary immunization activities, and routine vaccination, we generate the number of susceptibles in each age group in each year.

This method also outputs the estimated true incidence each year, meaning that we can both correct case numbers for underreporting and generate susceptibility profiles from the model output.

Using cases corrected for under-reporting to generate a canonical path

In addition to being used to generate estimates of susceptibility by age, the output of the Simons *et al.,* model gives us estimates of true measles incidence rather than reported incidence. These estimates can then be treated in the same way as the reported cases, to generate a trajectory through incidence-space for each country. The equivalent plots to Fig. 1A, 2, 3 and 4 for when cases are estimated are seen in Fig. S14, S26-S28.

Position and speed of movement along the canonical path

We calculated the position and speed of movement along the canonical path for each country between 1990 and 2017. We assigned countries to the nearest point on the granular version of the canonical path each year given their location in incidence space. We smoothed countries' position on the path over time by taking the average position for a given year along with two years before and after. Estimates begin in 1993, and 2016 and 2017 estimates are limited to 4 and 3 year averages, respectively. Speed of movement is simply calculated as the change in smoothed position from year to year. Yearly region averages (non-weighted and population-weighted (*23*)) were also calculated. Figure 3A-B display the results of this analysis. We additionally highlighted 10 countries interest that represent typical and non-typical movements by plotting their position and speed of movement over time in Fig. S17.

Direction of movement in incidence space

We estimated the observed and expected direction of movement for each country by year between 1990 and 2017 (total of 5044 observed movements). The observed direction of movement was estimated as the angle between a country's position in incidence space from year to year. We relied on our assignment of each country to the nearest point on the granular canonical path to estimate the expected direction of movement as the angle between the nearest point on the granular path to the point five positions further (representing a one year change); with the exception of the last 5 position on the granular path in which countries could move in the direction of the very last position. Angles ranging from -180 to +180 were grouped in 10 degree increments. We calculated frequencies of angles for each region in 10 and 8 year groupings (1990-1999, 2000-2009, and 2010-2017). We then smoothed the expected direction of movement frequencies for each region and year grouping by taking the average frequency for a given 10-degree angle group along with frequencies plus and minus 20 degrees. Figure 3C and 3D displays the results of this analysis.

We conducted an extensive supplemental analysis assessing direction of movement in incidence space by country and year compared to the canonical path. Figure S18 displays the difference between observed and expected direction of movement by country and year. We assigned both observed and expected movements to a quadrant (I-IV) and compared (Fig. S18A). We also directly compared direction of movement by subtracting the observed from expected and plotting the absolute differences on a color scale from green to brown (Fig. S18B). In order to assess

which dimension (incidence or cv) was contributing more to each movement, we transformed all movement vectors unto unit vectors and subtracted each observed unit vector dimension from the respective expected unit vector dimension (Fig. S18C-D).

The length of observed vectors was estimated and compared to the difference between observed and expected movements in order to assess the potential association between differences in observed and expected movements and the magnitude of the movement. We estimated quartiles and deciles of all observed vectors, and plotted them against absolute and quadrant differences in observed and expected movements (Table S1, Fig S19).

Distance from the canonical path

We estimated individual dimension (incidence and CV) distance and total distance from the canonical path of each country's position in incidence space between 1990 and 2017. The distance measures are divided by the total range of the canonical path for each dimension and multiplied by 100 to get percentage. The observed vector distance is divided by the total length of the canonical path times 100 to get percentage. Distance from the canonical path was grouped by distance per log population size of each respective country and year (Fig. S20), and by position on the canonical path (Fig. S21).

Generalized additive models

To fit the expected location in incidence-space given birth rate, vaccination level or susceptible recruitment (i.e., the number of susceptible individuals recruited into the population each year defined as birth rate multiplied by (1-vaccination coverage)), we fit a generalized additive model (*33*). Data is of the form $(Y_{i,} X_i), i = 1, \dots, n$ for a response variable Y and a predictor variable X (in general we may have a vector of predictor variables, but we consider only one predictor at a time). Here our response variable, Y, is either incidence or CV, and predictor variables, X, are one of birth rate, vaccination, or susceptible recruitment. The generalized additive model then fits the model,

$$E(Y|X) = s(X)$$

where s is an unknown smooth function, which was estimated from the data by the model. We repeated this for all six combinations of response and predictor variable. For each predictor variable, we plotted the expected incidence and CV for the range of values in our data, generating the estimates shown in Fig. S22 and Fig. S23 for reported cases and estimated cases corrected for under-reporting, respectively.

Supplementary Text: Direction of Movement Analysis

For all countries and years (1990-2016), the median difference between observed (1 year forward) and expected movement is 0.00 degrees (IQR -57.13 to 31.05 degrees), with a median absolute error of 39.00 degrees (IQR, 15.04 to 92.81 degrees). If we compare projected movement to movement 3 years forward, the median difference between observed and expected movement is -0.04 degrees (IQR, -57.56 to 26.30 degrees), and median absolute error is 36.19 degrees (IQR 14.40 to 88.66 degrees).

For all countries' movements between 1990 and 2016, 54.98% of observed movements were within the same quadrant of expected movement (Figure S18) and 54.10% were within +/- 45 degrees of the expected direction of movement (Figure S18-19). Figure S18 displays the difference between observed and expected movements by quadrant for each country and year. Given that the path was built from Americas and Africa regions, we would expect these regions to have more observed movements within expected quadrants. This was true; countries in the Americas and Africa regions moved within the expected (absolute) quadrant of movement 64% and 58% of the time, while 50%, 49%, 48%, and 55% of observed movements were within the expected quadrant in Eastern Mediterranean, Europe, South-East Asia, and Western Pacific regions, respectively.

Supplementary Text: Distance from Path Analysis

If we calculated distance relative to the length of the canonical path, the median distance of all positions (all countries positions from 1990 to 2017) from the canonical path was 0.083 (IQR, 0.037 to 0.154). The median distance in coefficient of variation, as a proportion of the span of the path in this dimension, of all positions from their projected position on the canonical path was -0.057 (IQR, -0.199 to 0.096). The median distance in incidence, as a proportion of the span of the path in this dimension, of all positions from the canonical path was -0.017 (IQR, -0.094 to 0.035).

There were 219 of 5044 "observations" for which the closest point on the path was the end of the path. For these 219 observations, 133 observations were moving towards (0,0), and distance from the path was forced to 0. The remaining 86 observations were moving in the positive direction in at least one dimension.

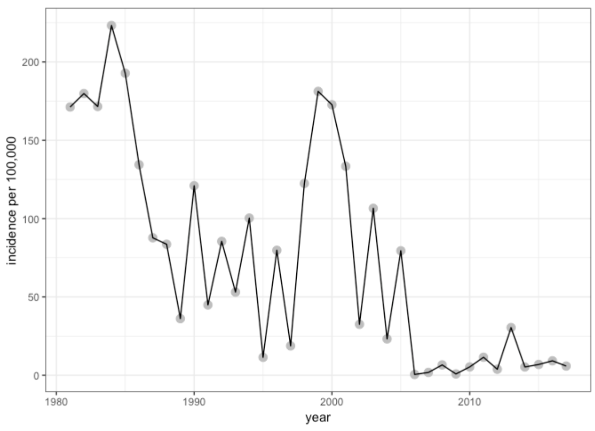

(A)

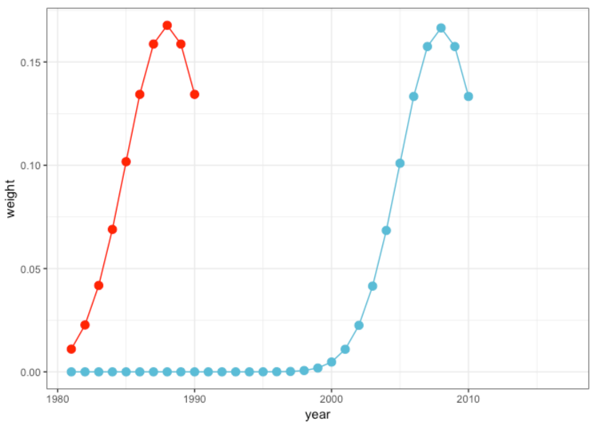

(B)

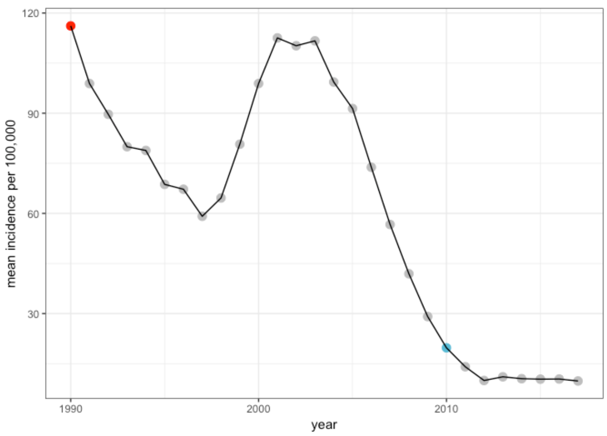

(C)

Fig. S1.
Example of calculating the weighted mean incidence over time. (A) Incidence per 100,000 from 1981-2014 for Nigeria. (B) Weighting functions which give us the values of weighted mean incidence in 1990 in red and 2010 in blue. (C) The weighted mean incidence given by multiplying incidence each year by the Gaussian function. Results for 1990 and 2010 are highlighted with the same colors as (B).

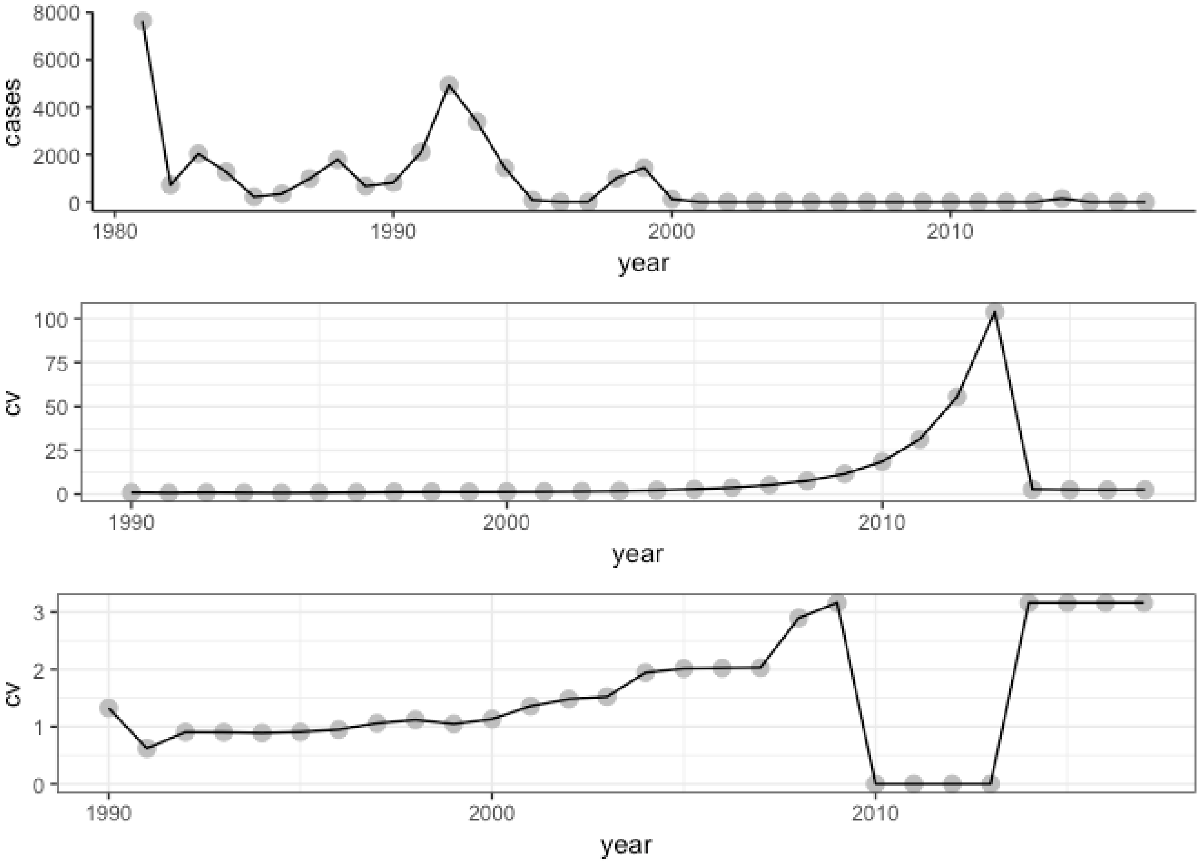

(A)

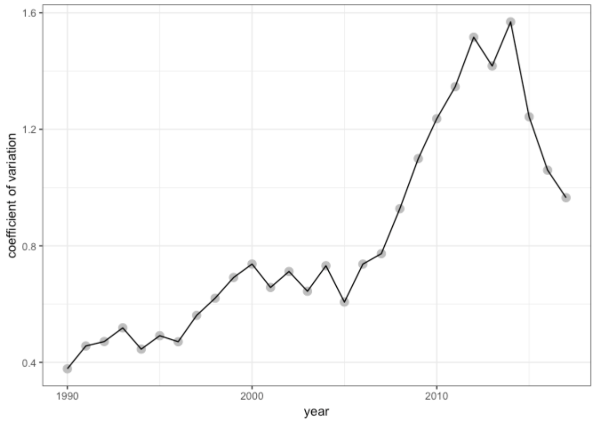

(B)

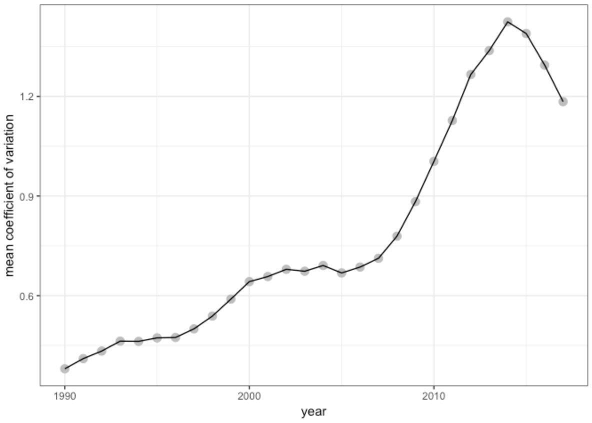

(C)

Fig. S2.
Examples of calculating the weighted coefficient of variation of incidence over time. (A) Shows cases by year for Bolivia, along with the result of using weighted standard deviation and weighted mean to calculate the CV over the whole period, and when we calculate unweighted "local CV" values over a period of 10 years. (B) Shows "local CV" for each year from 1990 to 2014 for Nigeria. (C) Shows the weighted mean of the "local CV" for Nigeria.

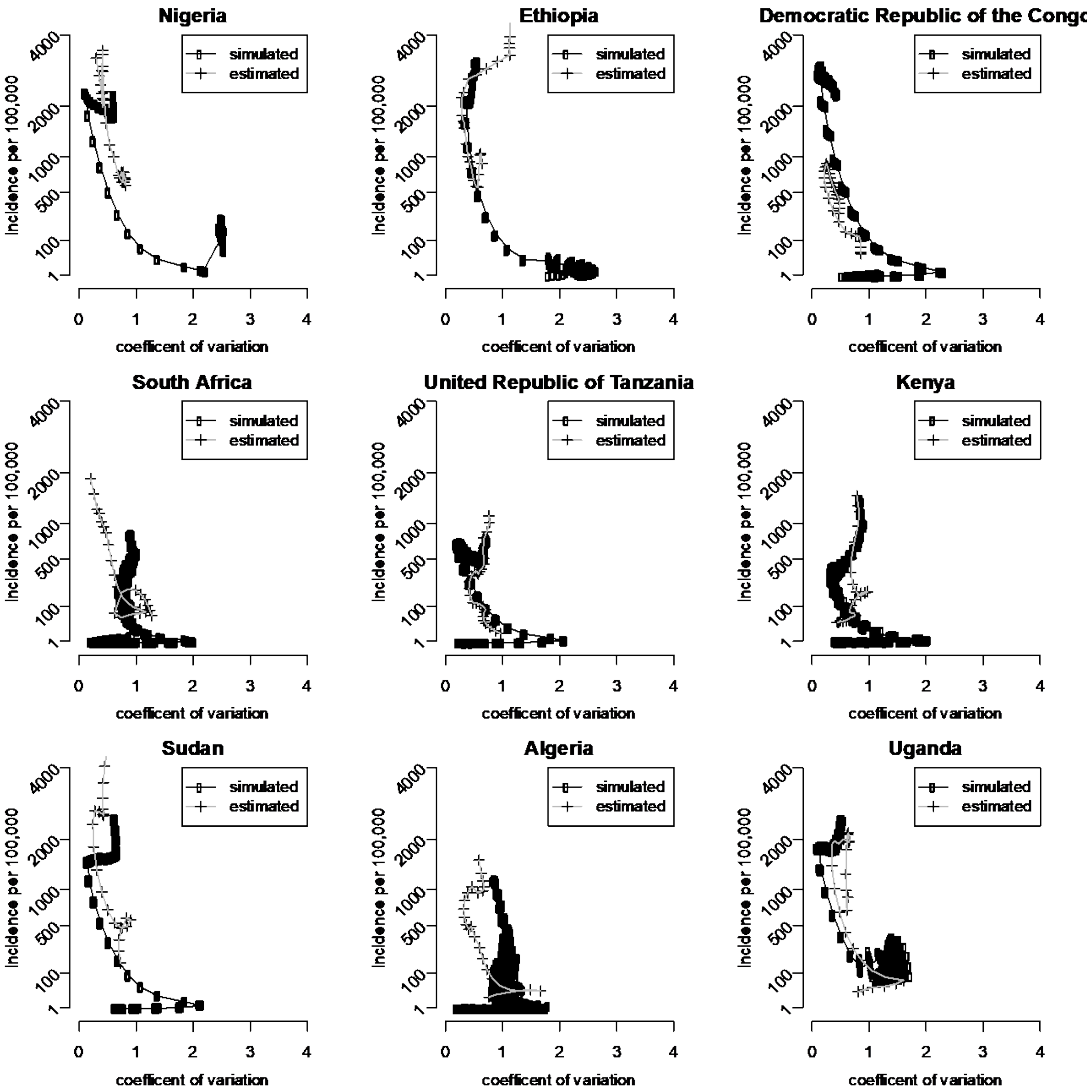

Fig. S3.

Simulated mean and coefficient of variation of measles incidence compared to estimated mean and CV of measles incidence for countries with population sizes exceeding 30 million from WHO's Africa Region, ordered by population size**.** The colored dots represent the simulated mean and coefficient of variation, and the plus signs represent the estimated mean and coefficient of variation. The colors from green to orange represent progression of the canonical path between 1990 and 2017, and black lines represent the means across all 100 simulations.

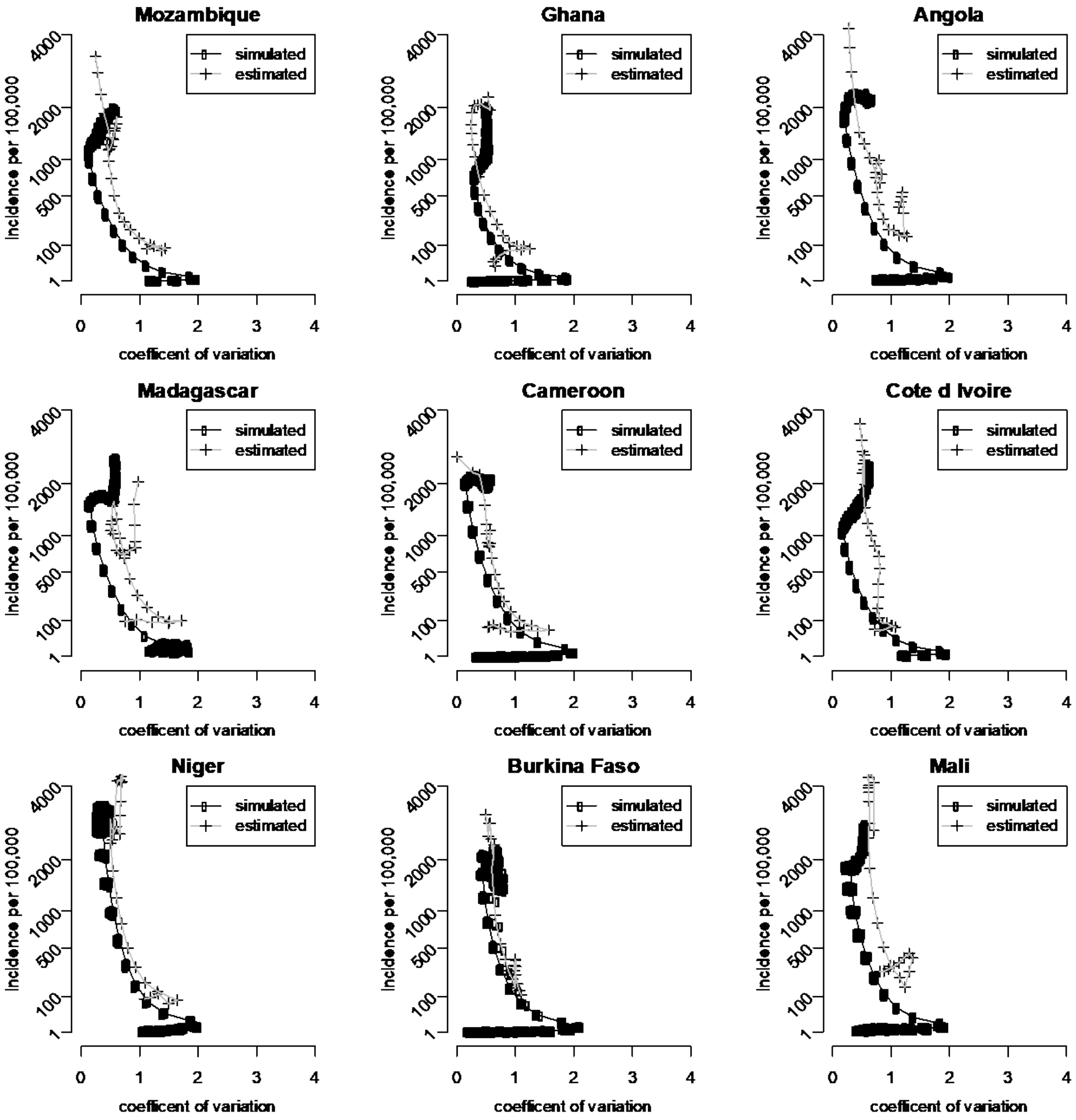

Fig. S4.
Simulated mean and coefficient of variation of measles incidence compared to estimated mean and CV of measles incidence for countries with population sizes between 25 and 15 million from WHO's Africa Region, ordered by population size**.** The colored dots represent the simulated mean and coefficient of variation, and the plus signs represent the estimated mean and coefficient of variation. The colors from green to orange represent progression of the canonical path between 1990 and 2017, and black lines represent the means across all 100 simulations.

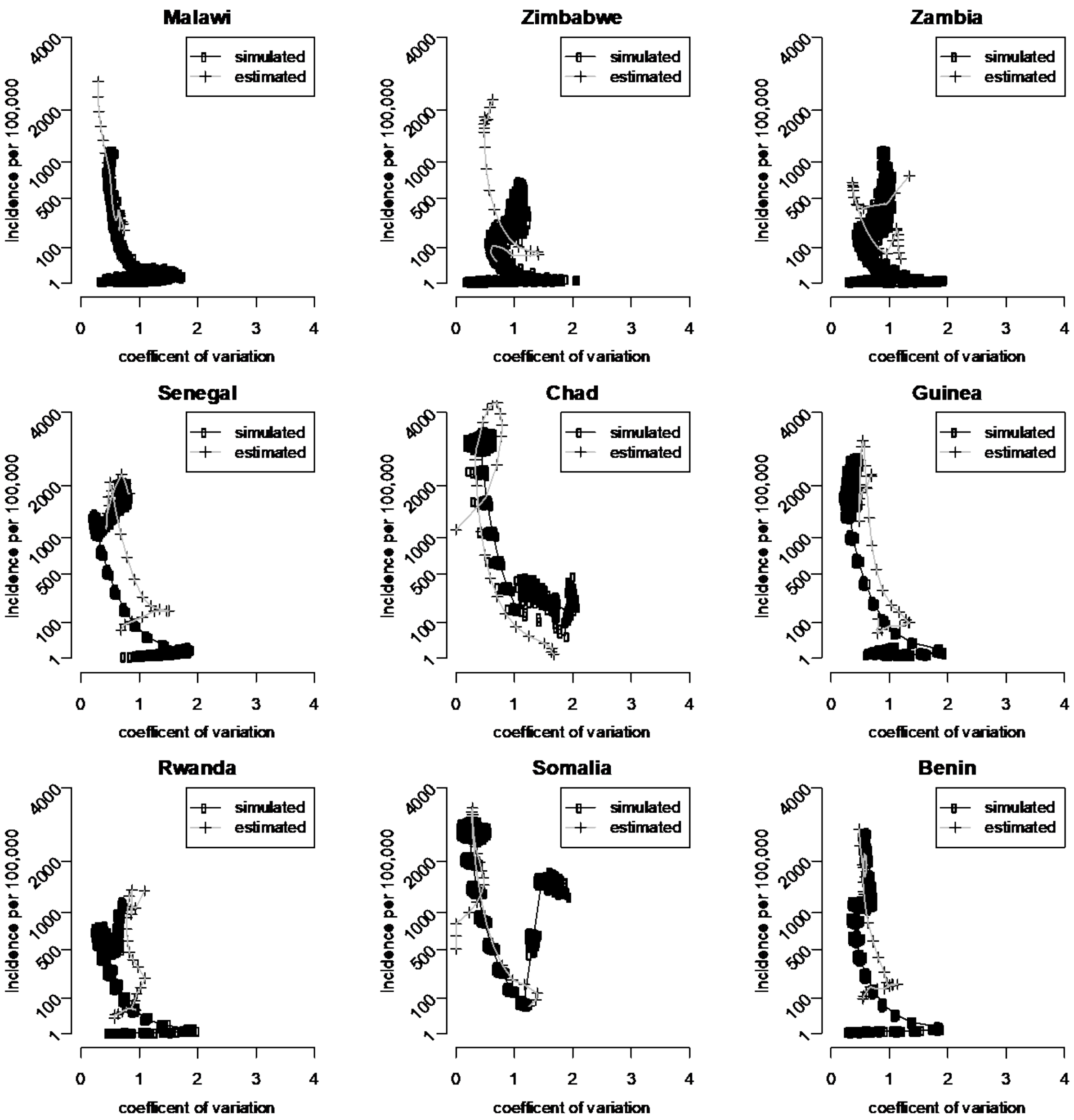

Fig. S5.
Simulated mean and coefficient of variation of measles incidence compared to estimated mean and CV of measles incidence for countries with population sizes between 14 and 9.5 million from WHO's Africa Region, ordered by population size. The colored dots represent the simulated mean and coefficient of variation, and the plus signs represent the estimated mean and coefficient of variation. The colors from green to orange represent progression of the canonical path between 1990 and 2017, and black lines represent the means across all 100 simulations.

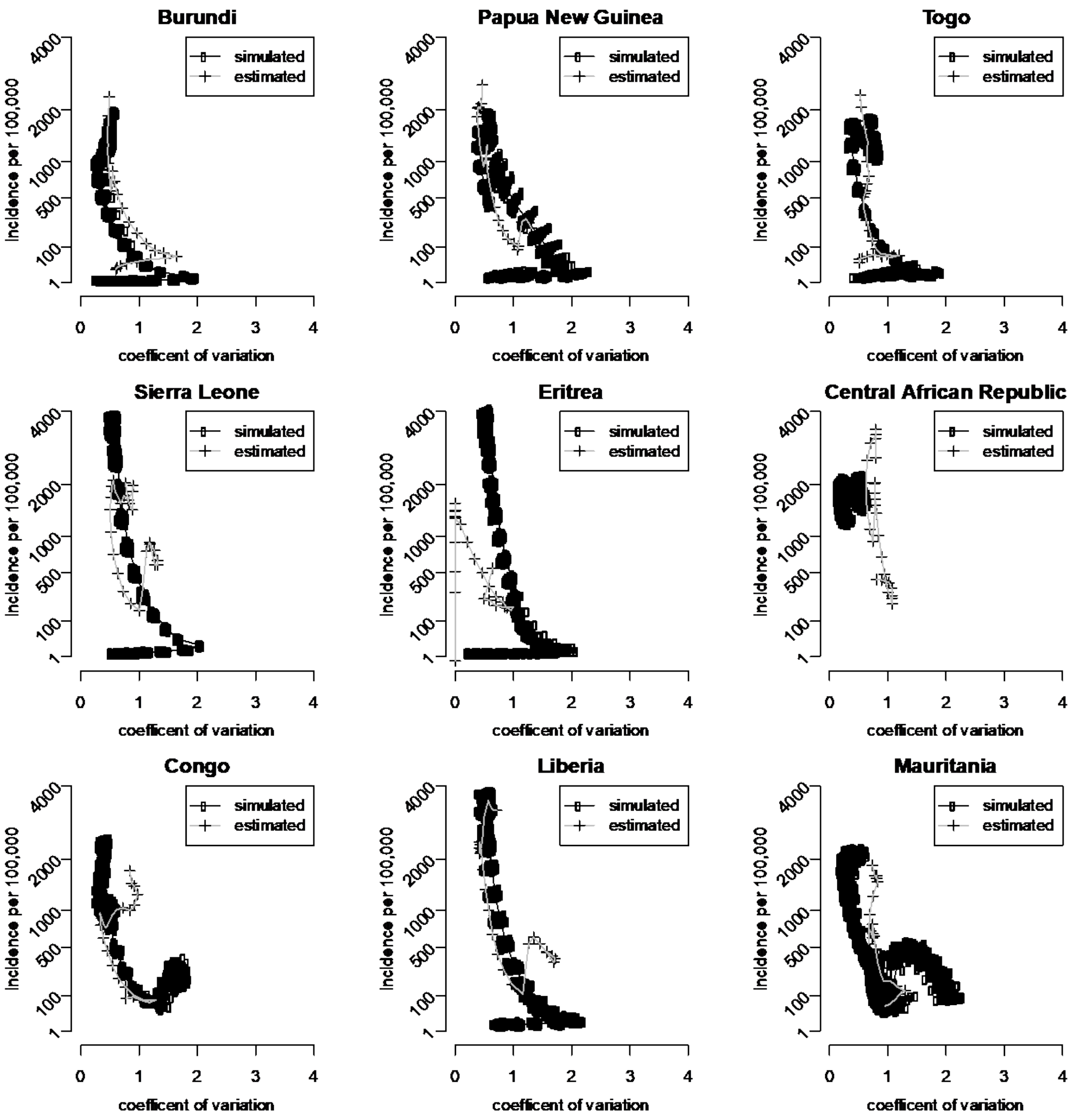

Fig. S6.

Simulated mean and coefficient of variation of measles incidence compared to estimated mean and CV of measles incidence for countries with population sizes between 9.4 and 3 million from WHO's Africa Region, ordered by population size**.** The colored dots represent the simulated mean and coefficient of variation, and the plus signs represent the estimated mean and coefficient of variation. The colors from green to orange represent progression of the canonical path between 1990 and 2017, and black lines represent the means across all 100 simulations.

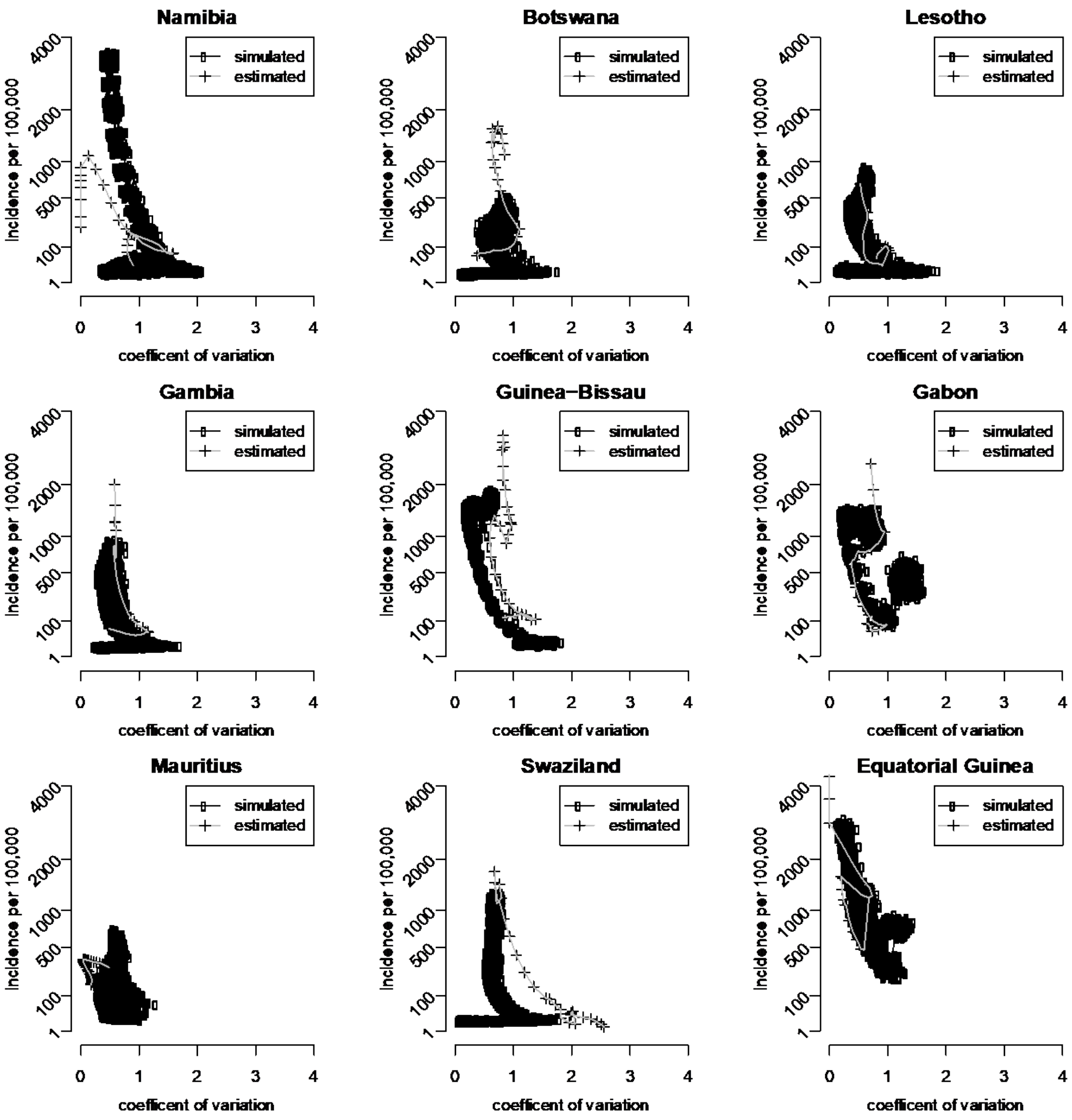

Fig. S7.

Simulated mean and coefficient of variation of measles incidence compared to estimated mean and CV of measles incidence for countries with population sizes between 2.2 million and 700,000 from WHO's Africa Region, ordered by population size. The colored dots represent the simulated mean and coefficient of variation, and the plus signs represent the estimated mean and coefficient of variation. The colors from green to orange represent progression of the canonical path between 1990 and 2017, and black lines represent the means across all 100 simulations.

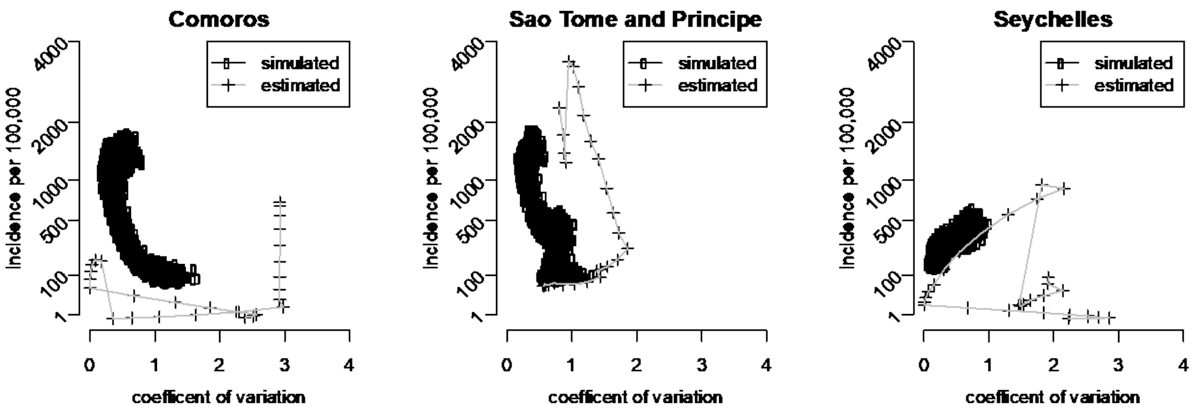

Fig. S8.
Simulated mean and coefficient of variation of measles incidence compared to estimated mean and CV of measles incidence for countries with population sizes less than 700,000 from WHO's Africa Region, ordered by population size**.** The colored dots represent the simulated mean and coefficient of variation, and the plus signs represent the estimated mean and coefficient of variation. The colors from green to orange represent progression of the canonical path between 1990 and 2017, and black lines represent the means across all 100 simulations.

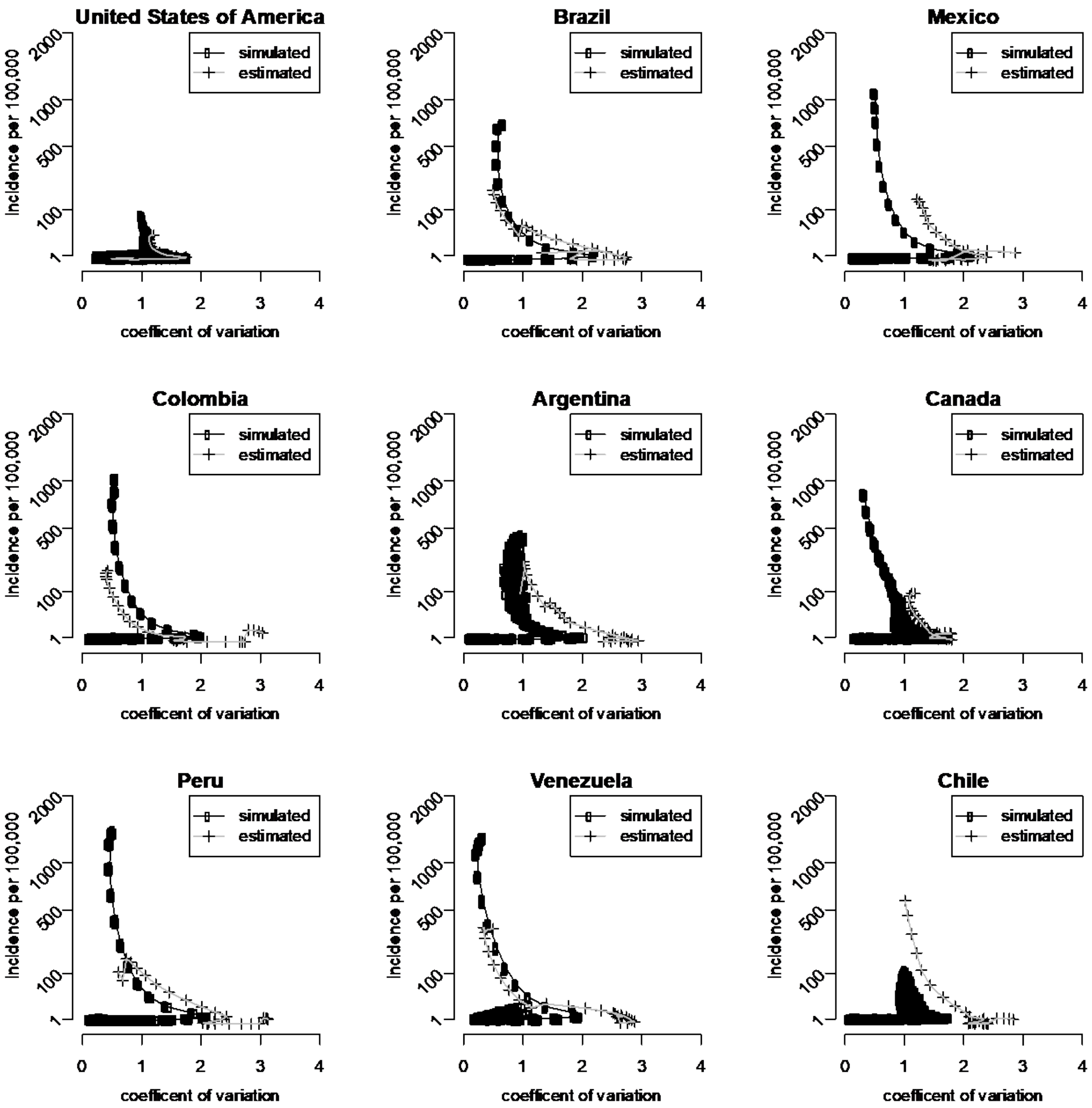

Fig. S9.
Simulated mean and coefficient of variation of measles incidence compared to estimated mean and CV of measles incidence for countries with population sizes exceeding 17 million from WHO's America Region, ordered by population size**.** The colored dots represent the simulated mean and coefficient of variation, and the plus signs represent the estimated mean and coefficient of variation. The colors from green to orange represent progression of the canonical path between 1990 and 2017, and black lines represent the means across all 100 simulations.

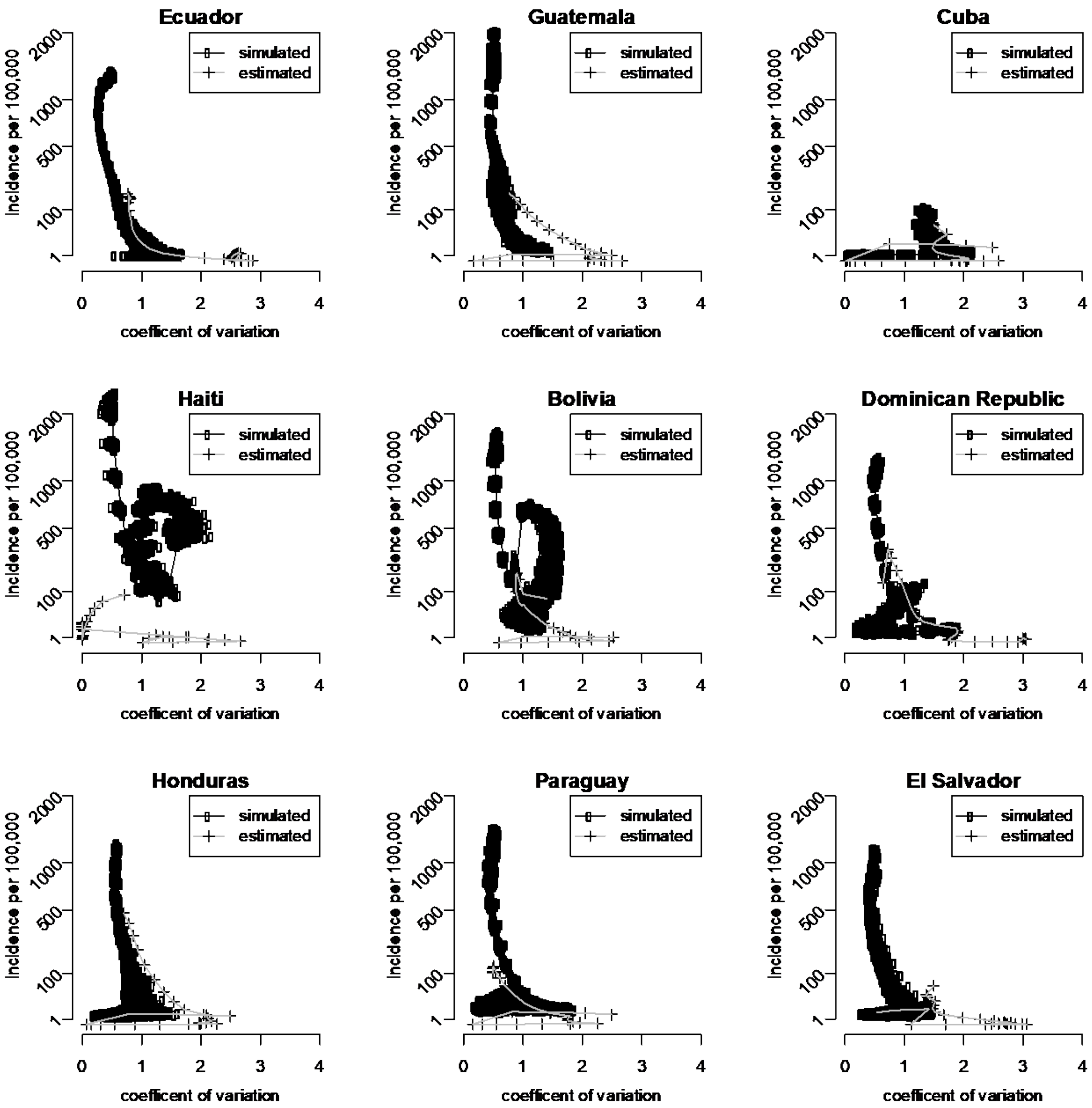

Fig. S10.
Simulated mean and coefficient of variation of measles incidence compared to estimated mean and CV of measles incidence for countries with population sizes between 15 and 6 million from WHO's America Region, ordered by population size**.** The colored dots represent the simulated mean and coefficient of variation, and the plus signs represent the estimated mean and coefficient of variation. The colors from green to orange represent progression of the canonical path between 1990 and 2017, and black lines represent the means across all 100 simulations.

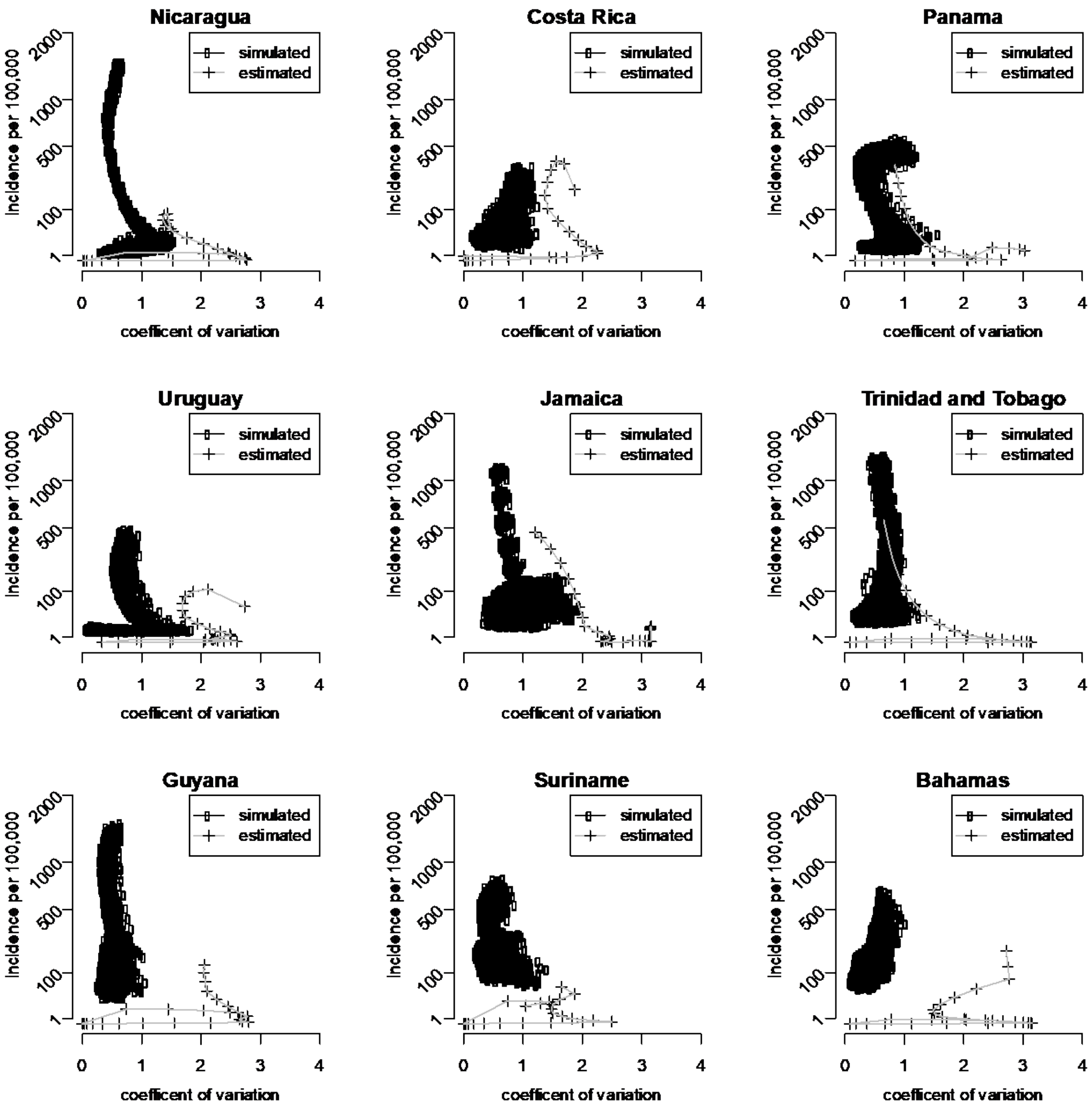

Fig. S11.
Simulated mean and coefficient of variation of measles incidence compared to estimated mean and CV of measles incidence for countries with population sizes between 5 million and 350,000 from WHO's America Region, ordered by population size. The colored dots represent the simulated mean and coefficient of variation, and the plus signs represent the estimated mean and coefficient of variation. The colors from green to orange represent progression of the canonical path between 1990 and 2017, and black lines represent the means across all 100 simulations.

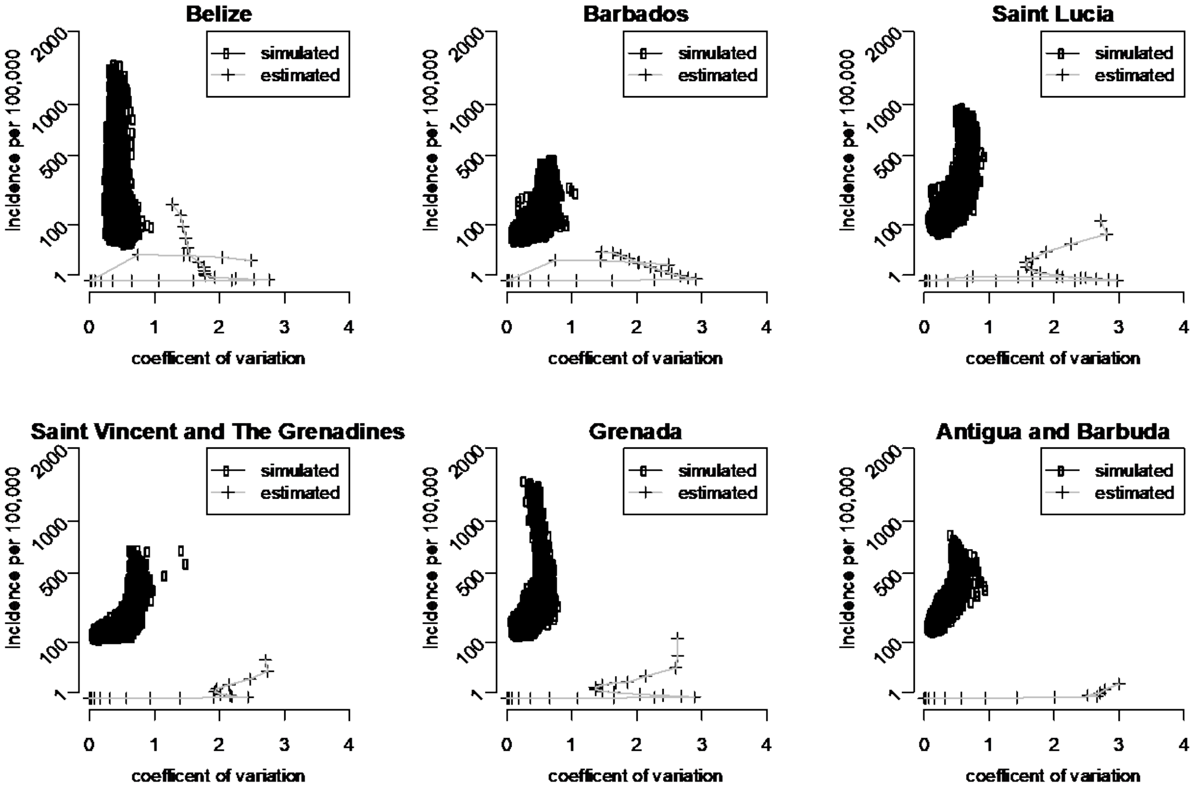

Fig. S12.
Simulated mean and coefficient of variation of measles incidence compared to estimated mean and CV of measles incidence for countries with population sizes less than 350,000 from WHO's America Region, ordered by population size**.** The colored dots represent the simulated mean and coefficient of variation, and the plus signs represent the estimated mean and coefficient of variation. The colors from green to orange represent progression of the canonical path between 1990 and 2017, and black lines represent the means across all 100 simulations.

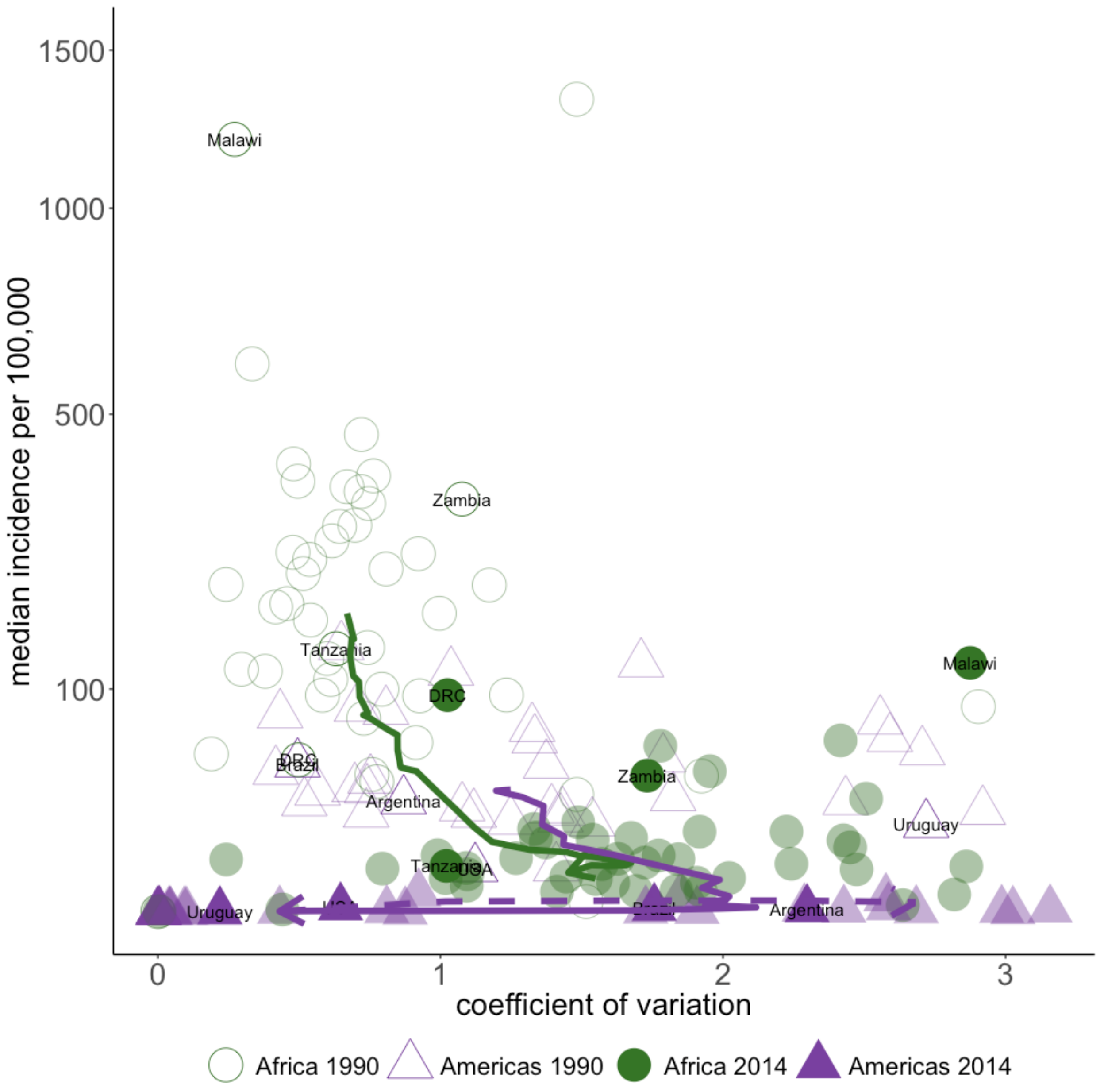

Fig. S13.
Characterizing the canonical path to elimination using median incidence. Similar to Fig. 1A in main text, but here the median is used to calculate the average position, rather than the mean as in Fig. 1. Dashed arrow shows the mean position of the Americas from 2015-2017.

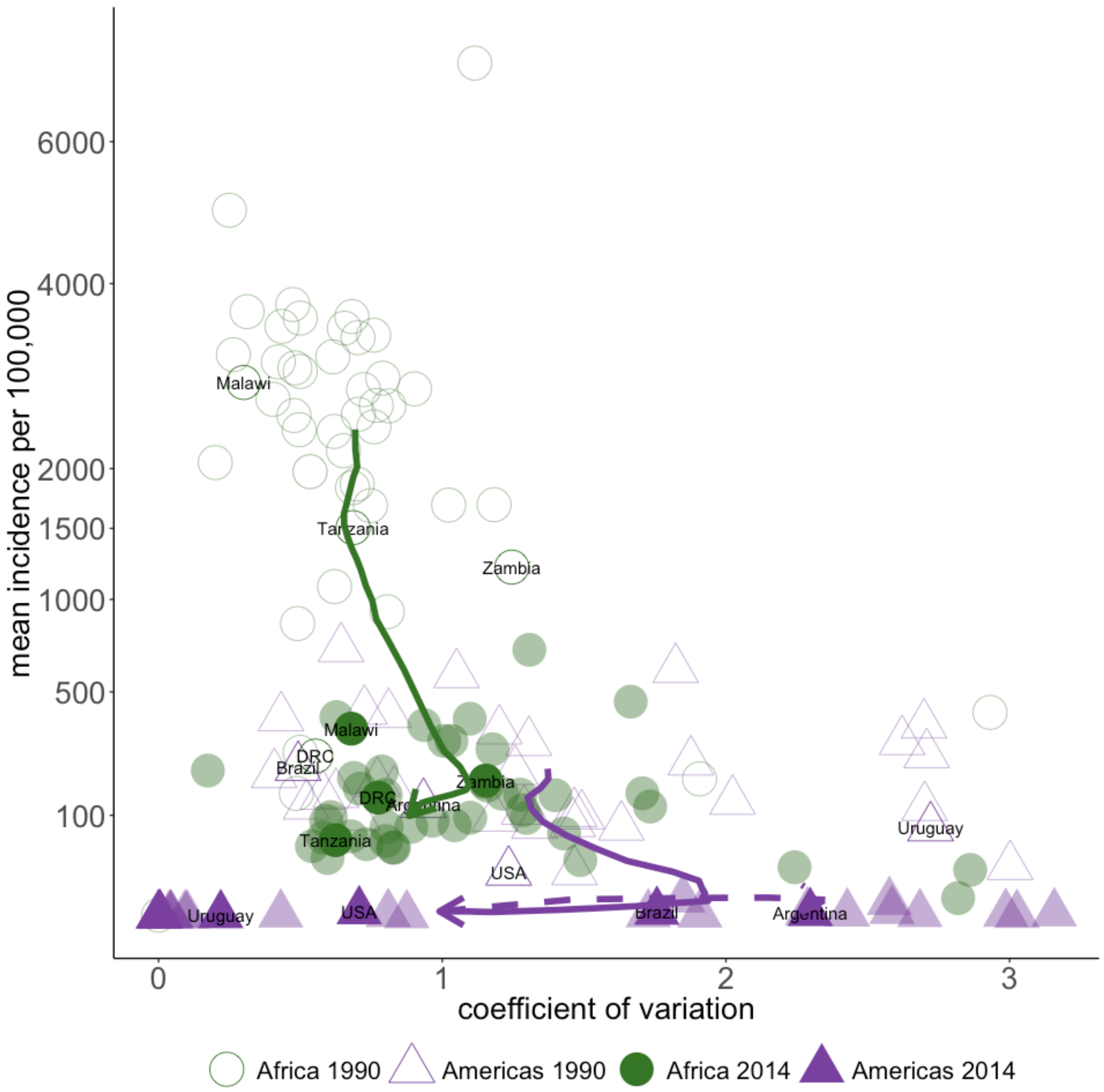

Fig. S14.
Characterizing the canonical path to elimination using incident cases corrected for under-reporting. Similar to Fig. 1A in main text, using estimated cases corrected for under-reporting rather than reported cases as in Fig. 1. Dashed arrow shows the mean position of the Americas from 2015-2017.

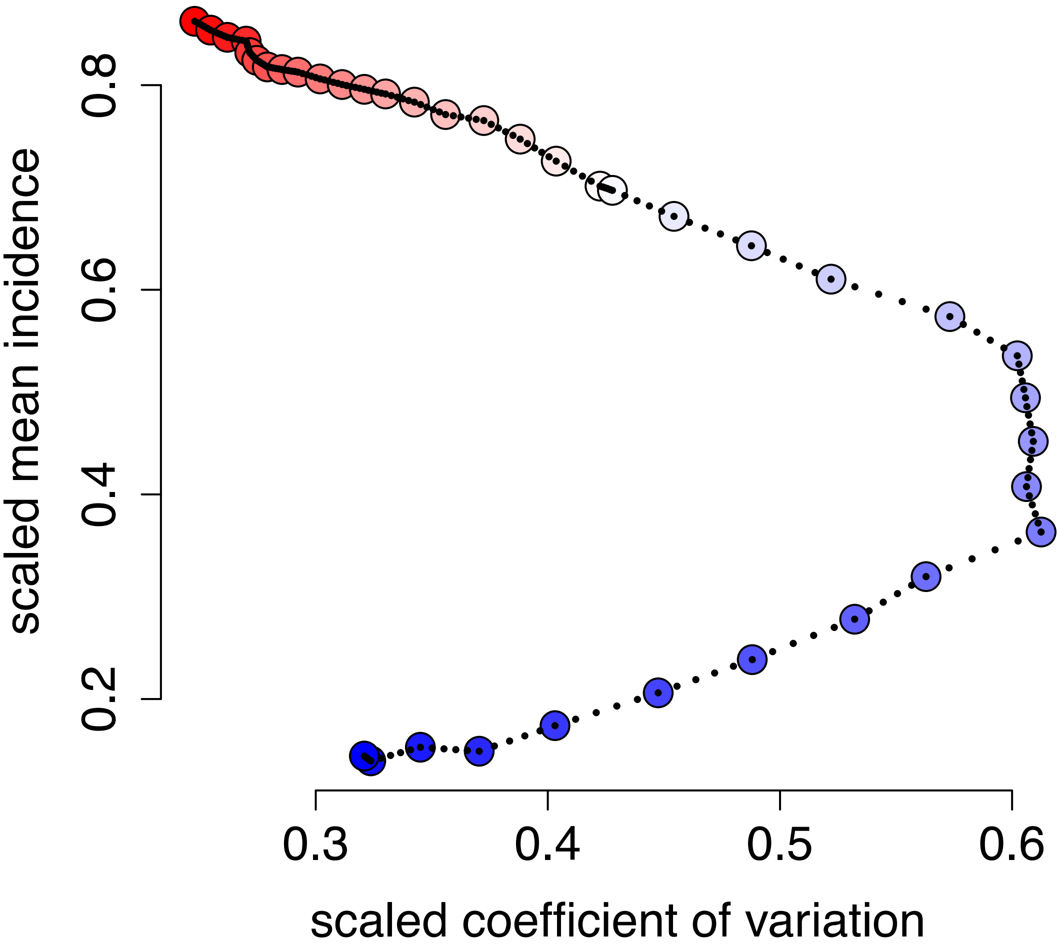

Fig. S15.
The canonical path along with a more granular version of the path, with equally spaced points. In color we see the original path scaled so that there is more resolution at the lower end of the incidence scale. The small black dots show a more granular version of the path, with the steps between the points all having the same size.

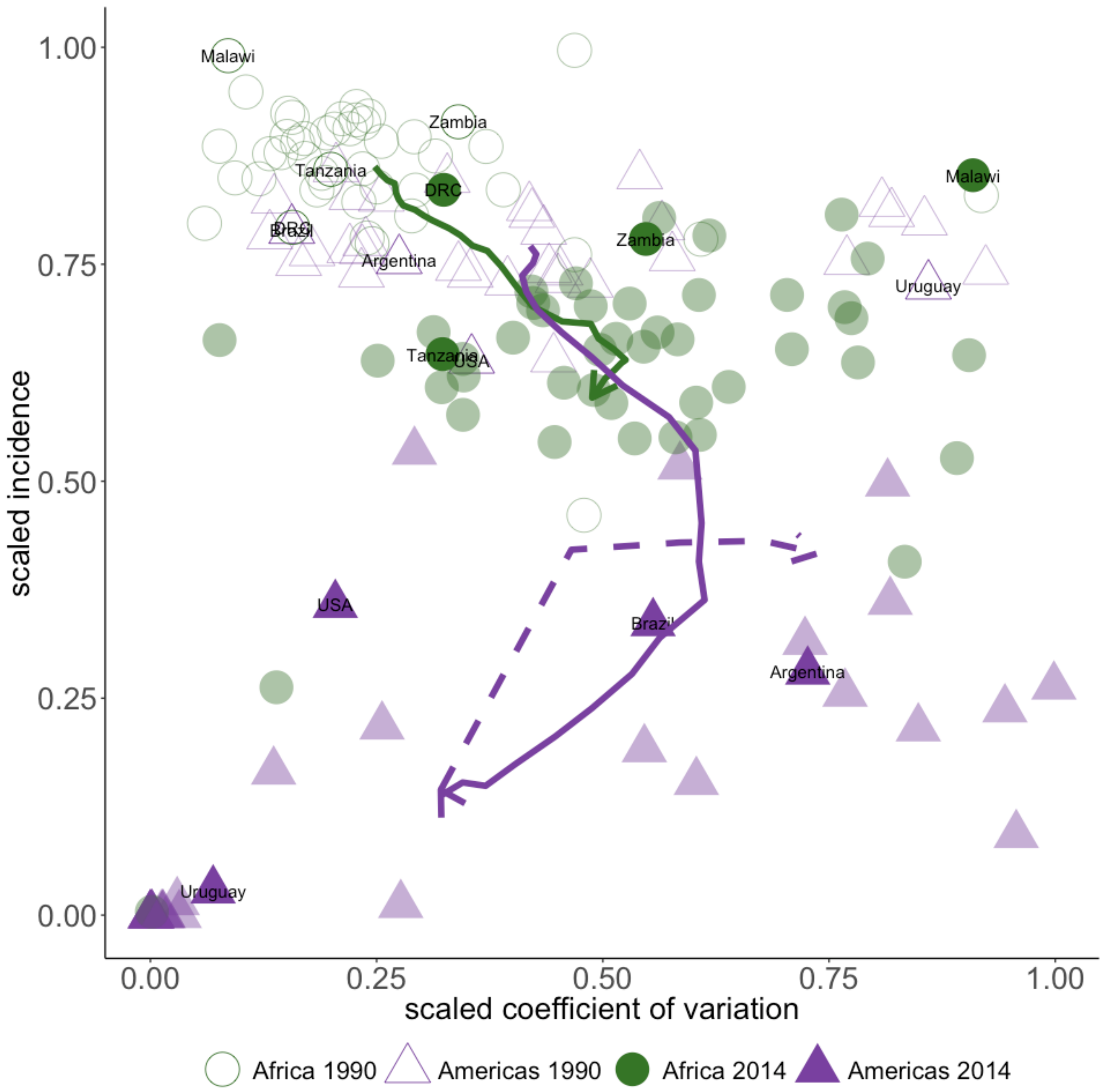

Fig. S16.
Location of countries in the WHO Africa region and region of the Americas in "incidence space" in 1990 and 2014 post scaling of incidence and CV. As countries position on x-axis increases, the unpredictability of the year-to-year cases increases. Arrows show the mean position of countries from these two WHO regions for each year in the intervening period. Dashed arrow shows the mean position of the Americas from 2015-2017.

(A)

(B)

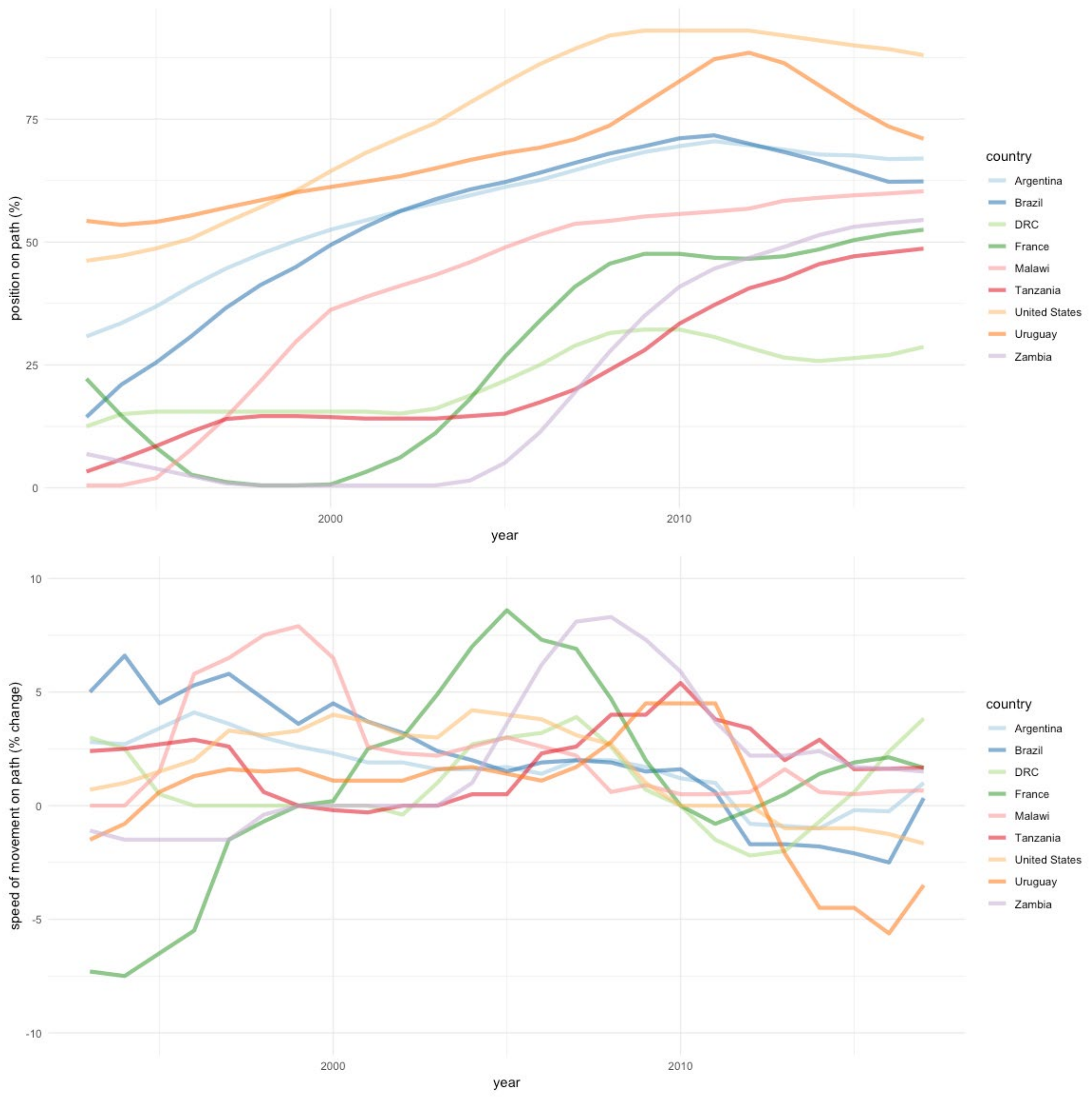

Fig. S17.
Movement along the canonical path for 10 countries of interest from the Americas, Africa, and European regions using reported measles cases. These 10 countries are simply lifted from Fig. 4A-B in the main text.

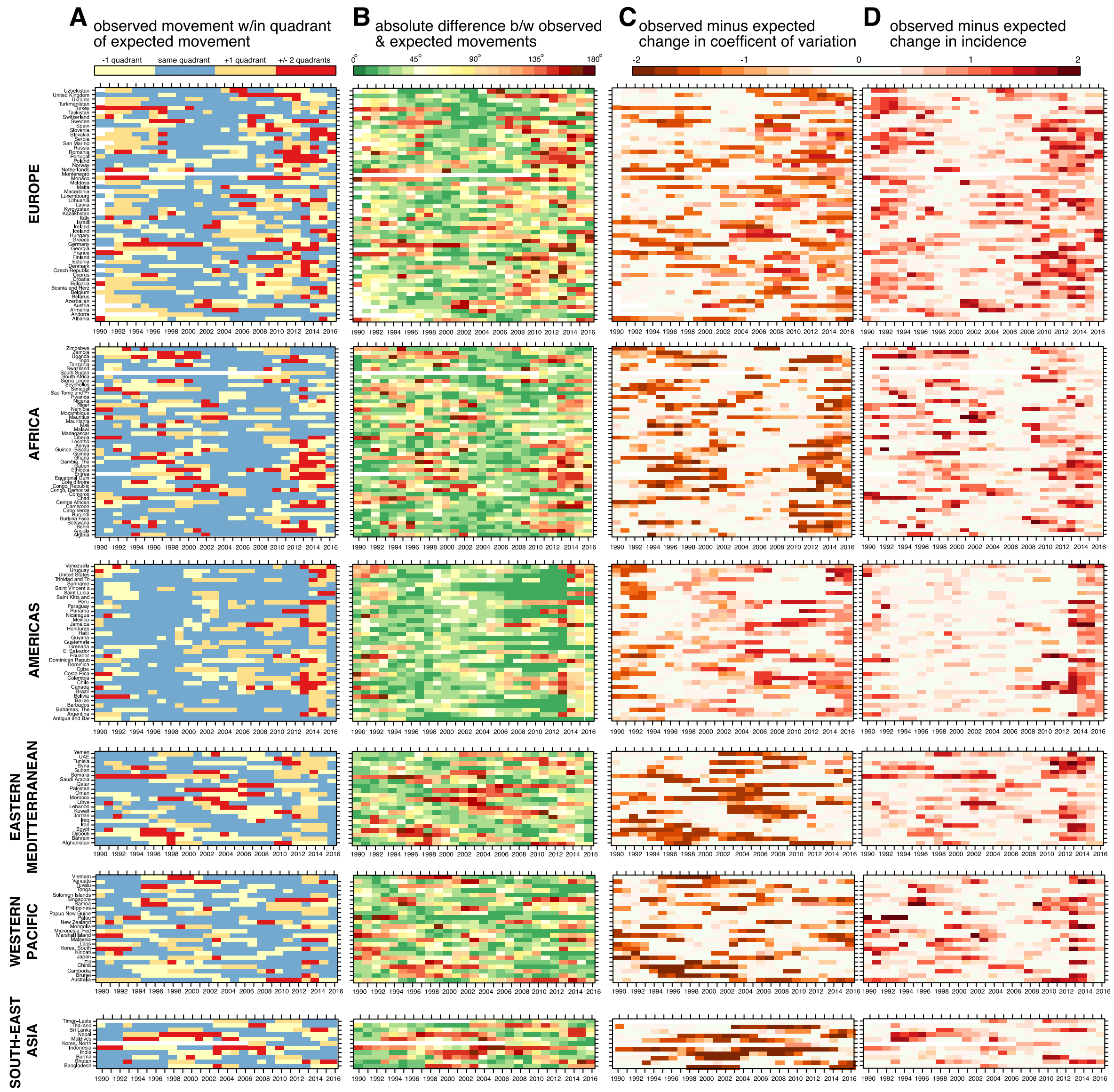

Fig. S18.

Difference between observed to expected direction of movements by country and year. Countries are in rows, grouped by WHO region, and years are in columns. **A)** Displays the difference between observed to expected quadrants of movements by country and year. The color blue means the observed movement occurred in the same quadrant as the expected movement. If the observed movement took place in 1 quadrant away clockwise from the quadrant of the expected movement, then represented by pale yellow (-1 quadrant). If the observed movement took place in 1 quadrant away counter-clockwise from the quadrant of the expected movement, then represented by pale tan (+1 quadrant). If the observed movement took place +/- 2 quadrants away from the quadrant of the expected movement, then represented by red (+/-2 quadrant). **B)** Displays absolute difference between observed and expected direction of movements by country and year. Tints of green represent when observed movement was within +/- 45 degrees of expected movement, as colors move from yellow to orange to red observed movements are further and further away from expected movements. **C-D)** Compares observed from expected movements independently for the coefficient of variation (C) and incidence (D) axes.

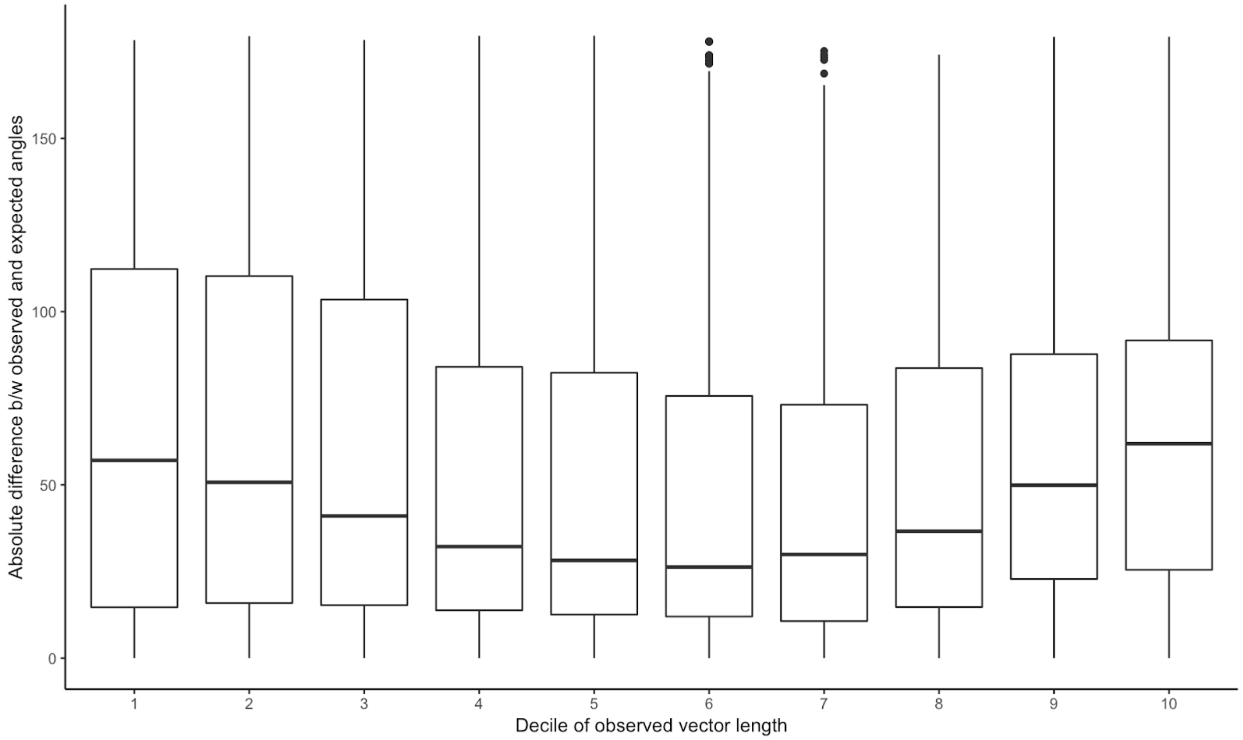

Fig S19.
Absolute difference between observed and expected movements by observed vector length (deciles). N = 5044 total movements for countries between 1990 and 2017.

Table S1.
The number (proportion) of observed movements within quadrants of expected movement per quartiles of observed vector lengths, 1990 to 2017. There are 5044 total movements for countries between 1990 and 2017. The observed movement either took place one quadrant clockwise away from expected quadrant of movement (-1 quadrant), in the same quadrant as expected quadrant of movement, one quadrant counter-clockwise from expected quadrant of movement (+1 quadrant) or two quadrants away from expected quadrant of movement (+/- 2 quadrants).

Observed Vector Length Quartiles	-1 quadrant	Same quadrant	+1 quadrant	+/- 2 quadrants	Total
0-25th	331 (0.262)	600 (0.476)	164 (0.130)	166 (0.132)	1261 (1.00)
25-50th	252 (0.200)	753 (0.597)	135 (0.107)	121 (0.096)	1261 (1.00)
50-75th	201 (0.159)	788 (0.625)	162 (0.128)	110 (0.087)	1261 (1.00)
75-100th	222 (0.176)	632 (0.501)	310 (0.246)	97 (0.077)	1261 (1.00)

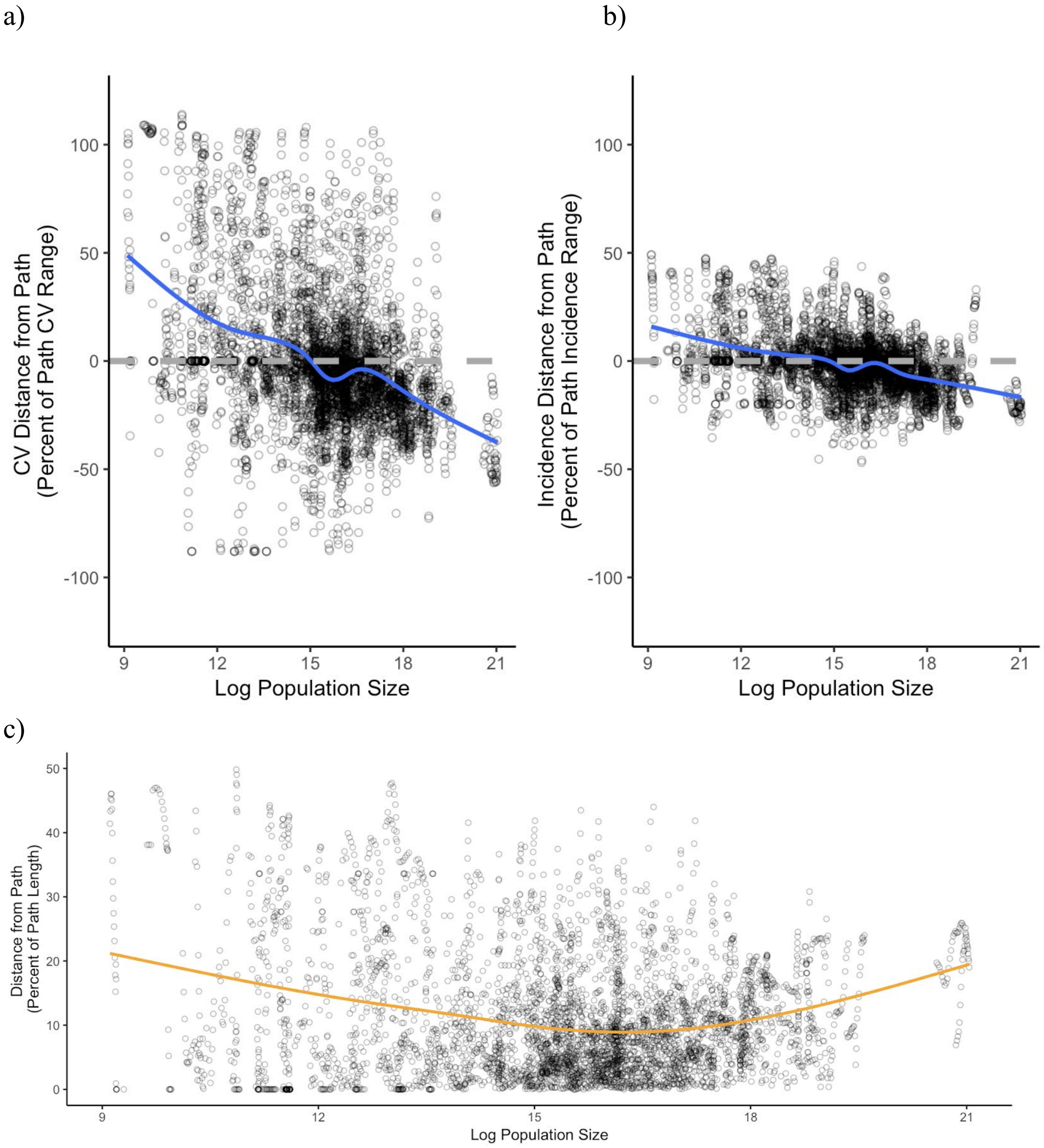

Fig S20.
Distance from the canonical path by log population size. Coefficient of variation (a) and incidence (b) distance of each county and year's position in incidence-space to the closest point on the path by log population size. The distance measures are divided by the total range of the canonical path for each dimension (cv or incidence) and multiplied by 100 to get percentage. c) Vector distance of each country and year's position in incidence-space to the closest position on the path by log population size. The vector distance is divided by the total length of the canonical path times 100 to get percentage. Each point represents a country and year position, the smoothed lines are fitted with splines.

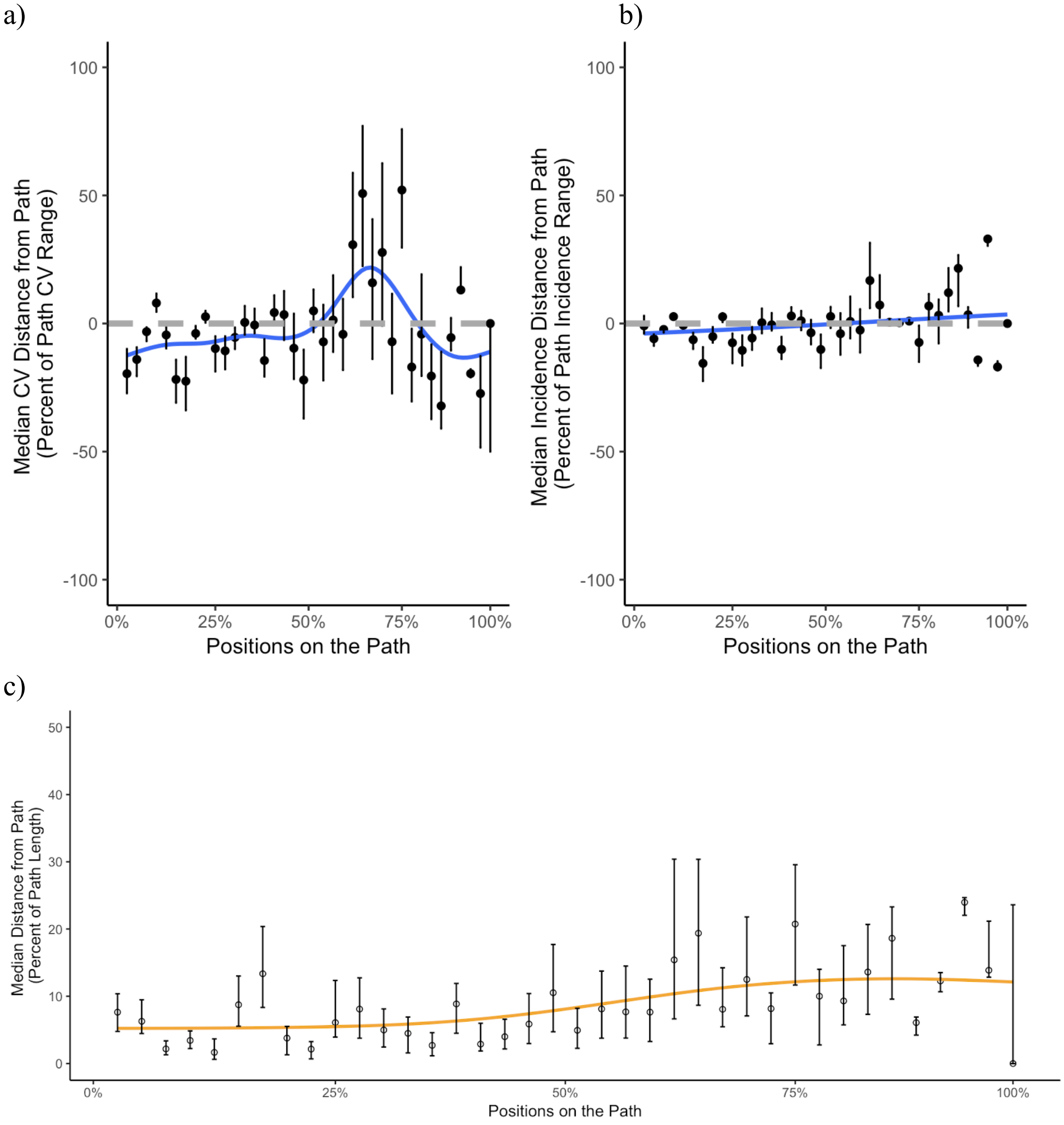

Fig S21.
Distance from canonical path by position on the canonical path. Coefficient of variation (a) and incidence (b) median distance of all positions in incidence-space to the closest point on the path by each canonical path position. The distance measures are divided by the total range of the canonical path for each dimension (cv or incidence) multiplied by 100 to get percentage. c) Vector distance of all positions in incidence-space to the closest point on the path by each canonical path position. The vector distance is divided by the total length of the canonical path times 100 to get percentage. The points and error bars represent the median and interquartile range, the smoothed lines are fitted with splines.

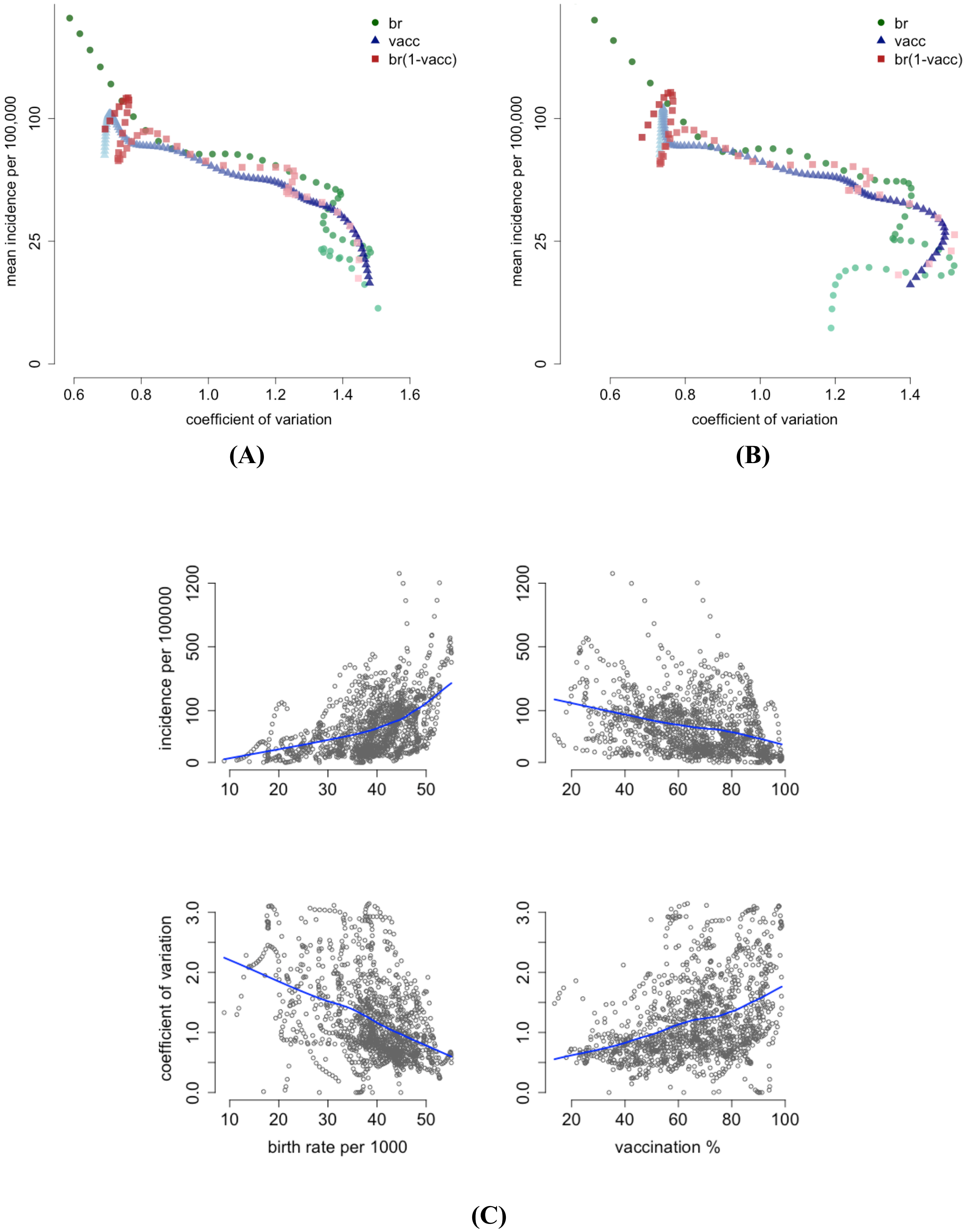

br
vacc
br(1-vacc)
mean incidence per 100,000
coefficient of variation
(A)
(B)
incidence per 100000
coefficient of variation
birth rate per 1000
vaccination %
(C)

Fig. S22.

Expected mean and coefficient of variation of incidence given a range of birth rates, vaccination coverage levels, and susceptible recruitment. Output is based on a statistical generalized additive model using reported cases (in comparison to Fig. S23 which uses estimated cases corrected for underreporting). Values ranged for birth rate per 1,000 people (7 to 56; br, green points), vaccination coverage (0 to 99%; vacc, blue triangles), and susceptible recruitment per 1,000 (0 to 48; br(1-vacc), red squares). Generalized additive models were fit to data from: (A) all regions combined, and (B) all regions excluding Europe. Generally, over time we moved toward the lower right of the figures A and B, with birth rate and susceptible recruitment decreasing and vaccination coverage increasing. (C) Association between incidence or coefficient of variation with the birth rate and vaccination coverage in Africa. We recovered the expected positive correlation between birth rate and incidence and the negative correlation between vaccination and incidence. When birth rates decreased, variation increased, as with lower birth rates we recruited fewer susceptibles into the population, reducing the predictability of dynamics. Similarly increasing vaccination coverage diminished susceptible recruitment, and hence drove bigger uncertainty in the dynamics.

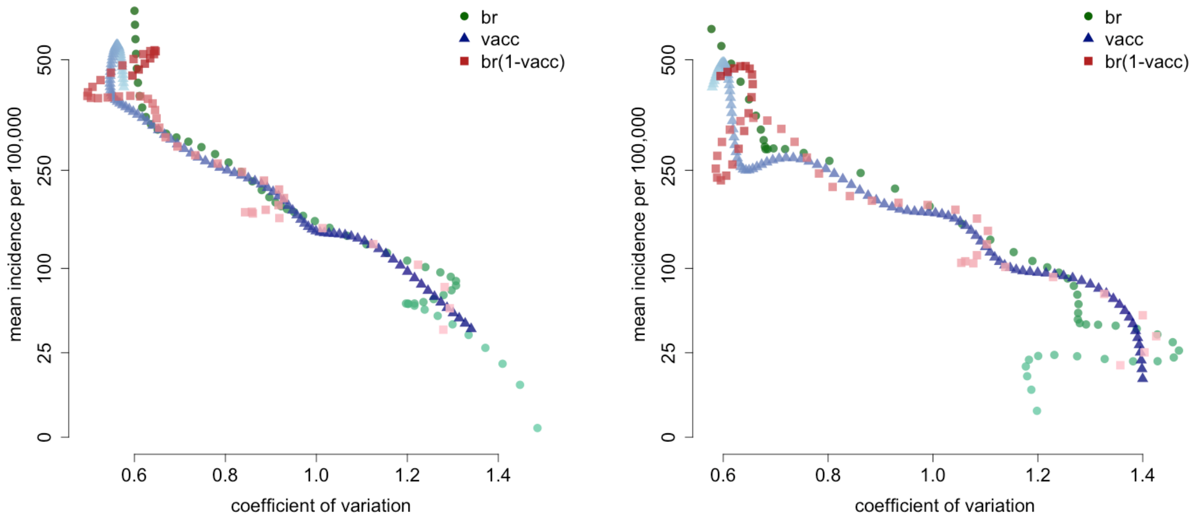

(A) **(B)**

Fig. S23.
Expected mean and coefficient of variation of incidence given a range of birth rates, vaccination coverage levels, and susceptible recruitment. Output is based on a statistical generalized additive model using estimated cases corrected for underreporting (in comparison to Fig. S22 which uses reported cases). Values ranged for birth rate per 1,000 people (7 to 56; br, green points), vaccination coverage (0 to 99%; vacc, blue triangles), and susceptible recruitment per 1,000 (0 to 48; br(1-vacc), red squares). Generalized additive models were fit to data from: (A) all regions combined, and (B) all regions excluding Europe. Generally, over time we moved toward the lower right of the figure, with birth rate and susceptible recruitment decreasing and vaccination coverage increasing.

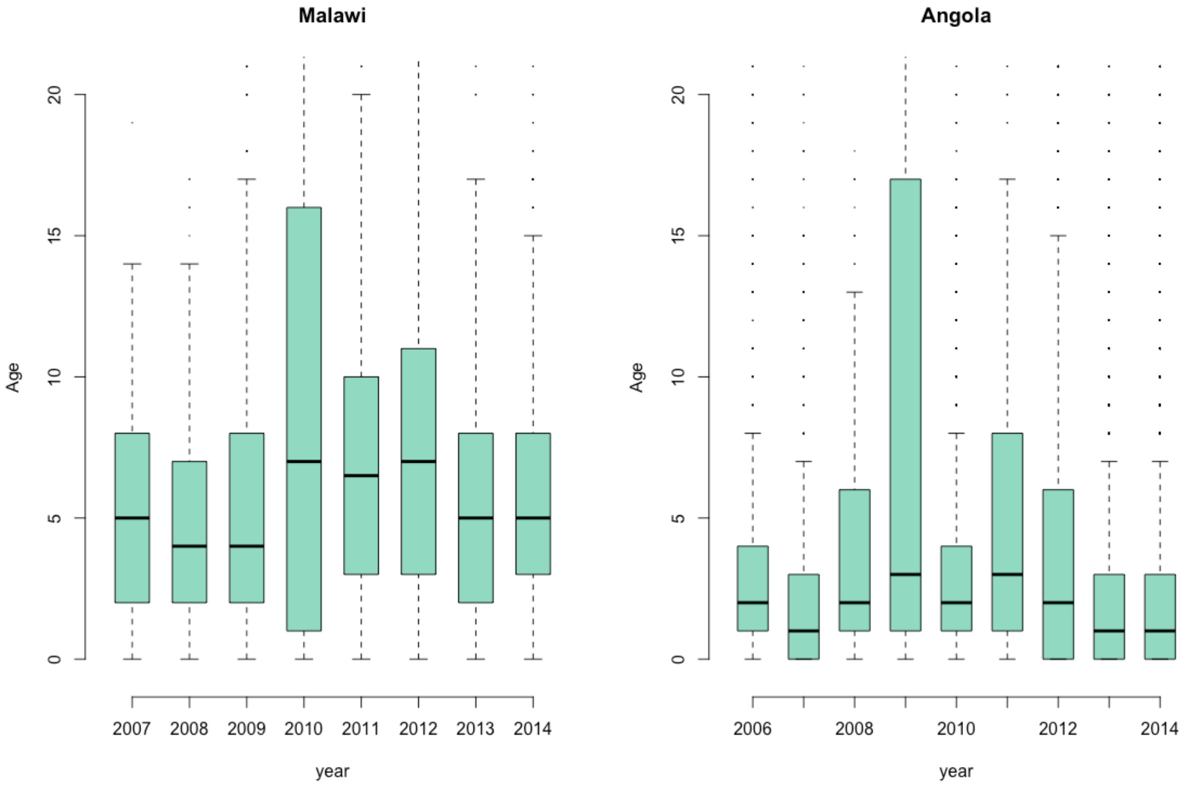

Fig. S24.
Age distribution of cases in Malawi and Angola. The y-axis is shown only up to age 20, but cases also occur in older individuals. Outbreaks with many more cases in older individuals than in previous years were seen in Angola in 2009 and Malawi in 2010. In the case of Malawi, this outbreak was also far larger than those in recent years.

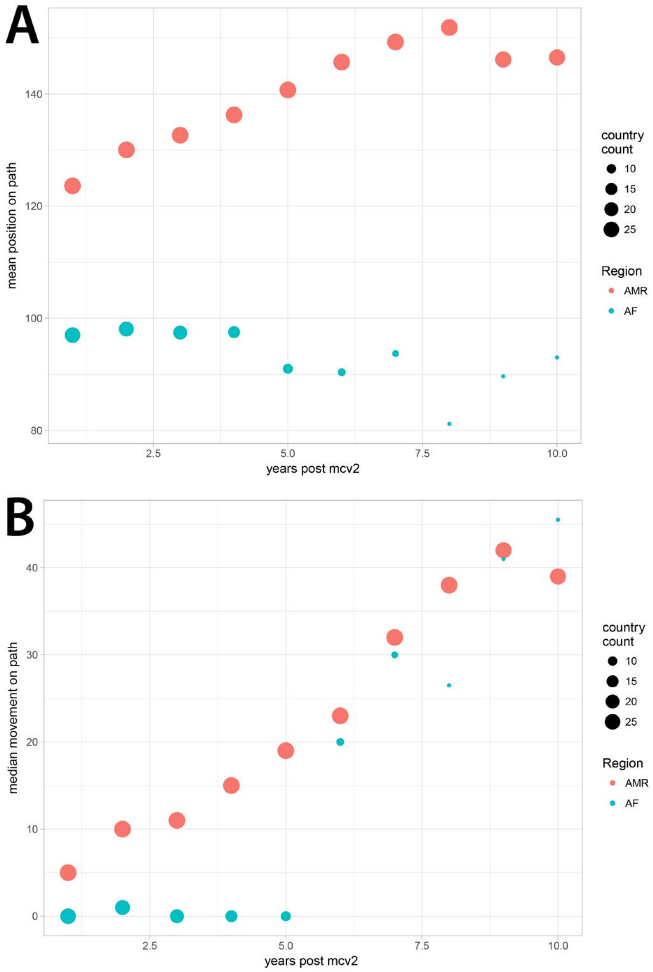

Fig. S25. Comparison of (A) path position and (B) speed of movement of countries from the WHO Africa and Americas regions by years since MCV2 introduction.

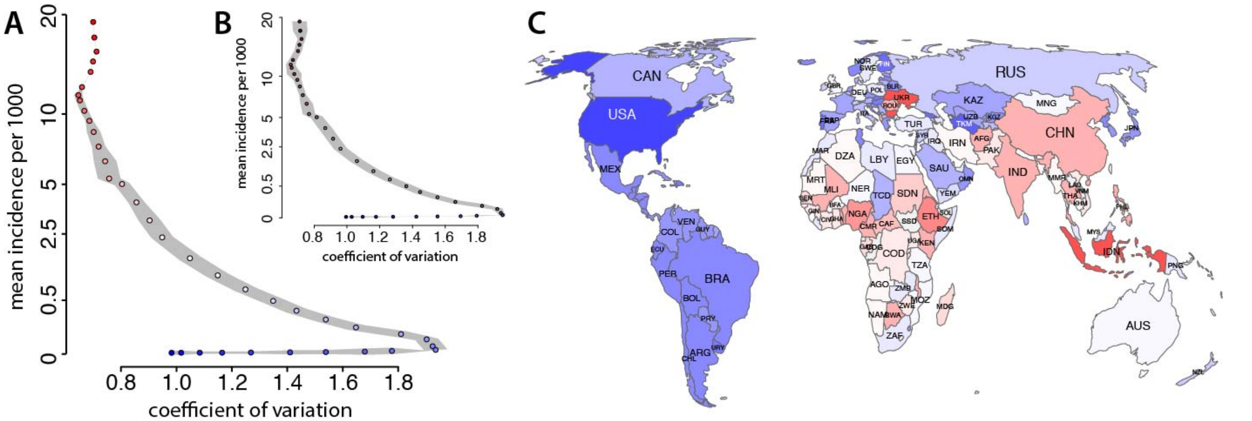

Fig. S26
Progression along the path and the age of susceptibility using estimated measles cases corrected for underreporting. In Fig. S28, the position along the canonical path is indicated by the corresponding color as shown in (A, B). Global population distribution given each country's further position on the path between 2012 and 2017 (A) and each country's earliest position on the path (B). Thickness of line represents the total population at each point on the canonical path. (C) Shows each country's position along the path as of 2017.

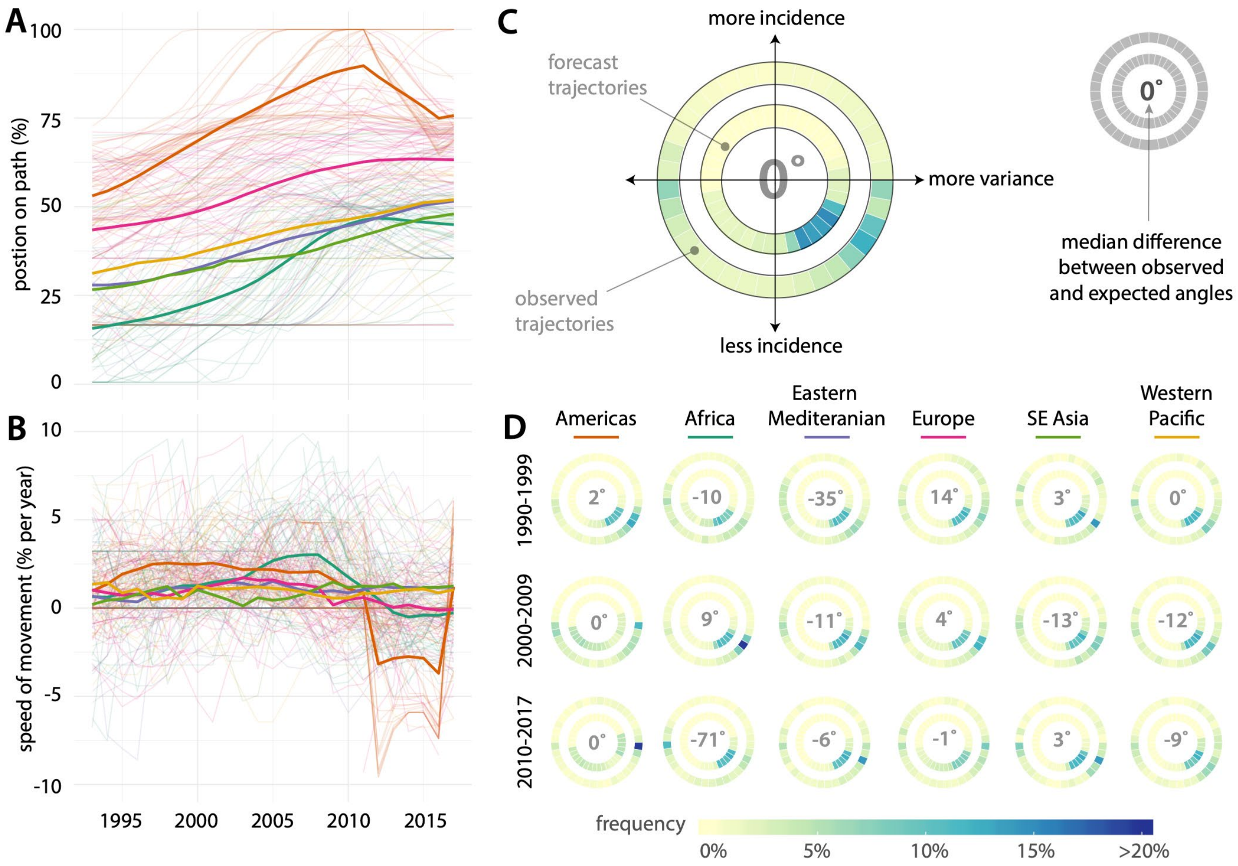

Fig. S27.
Analysis movement along the canonical path using estimated measles cases corrected for underreporting. A) The position of each country (light lines) and mean position of each WHO region (heavy lines) along the canonical path, measured as percent complete. (B) The speed of movement along the path as percent per year. (C) Heat map of the predicted angle of movement in incidence space based on the projected position of each country onto the canonical path in each year (inner circle), versus the actual observed angles of movement (outer circle). Bluer shades represent a higher proportion of countries moving (or being predicted to move) that direction. The inner number shows the median difference between the predicted and observed angles of movement across all countries and years. (D) Predicted and observed angles of movement among all countries in each WHO region in each decade period since 1990.

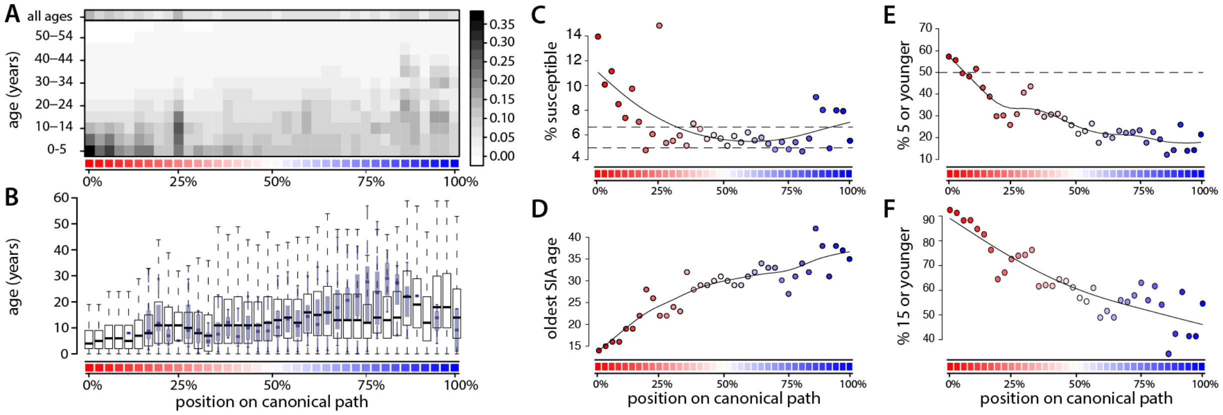

Fig. S28.

Progression along the path and the age of susceptibility using estimated measles cases corrected for underreporting. In all plots, the position along the canonical path is indicated by the corresponding color as shown in Fig 2A and percentage complete. (A) Age-specific proportion susceptible for each position on the canonical path. The legend on the right side of the figure, correlated with the colors in the plot, represent the estimated age-specific proportion susceptible. (B) The mean age of susceptibility to measles estimated by our approach (hollow wide boxes) and the mean age of cases for countries reporting this data around the world from 1995-2016 (filled narrow boxes) by canonical path position. (C) The proportion of susceptibles who are age 5 years or younger. (D) The oldest age that would need to be targeted by an SIA to cover 90% of the susceptible population.

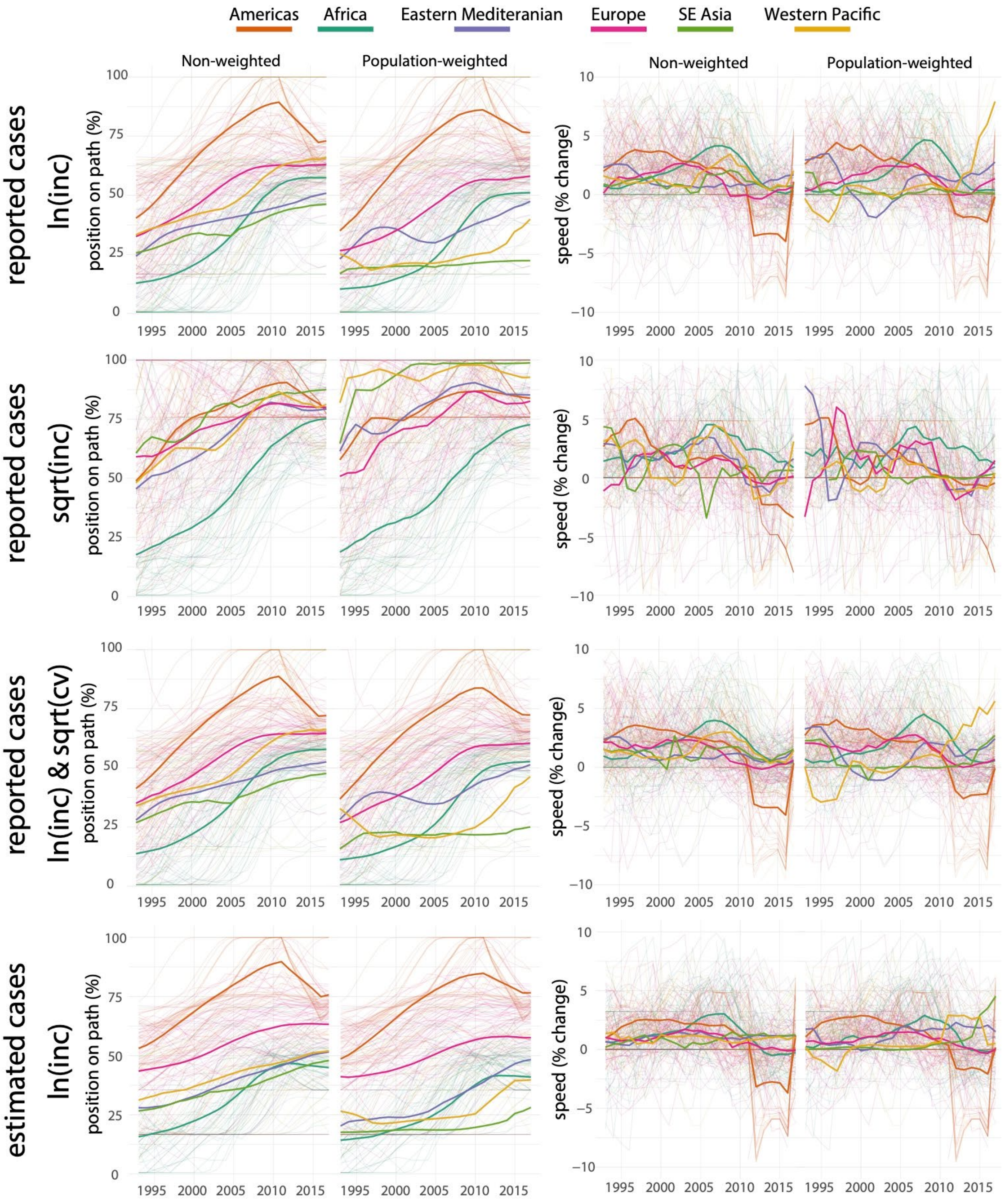

Fig. S29.

Sensitivity analysis of incidence space projections on position and speed of movement analysis. The first rows represent three different projections assessed, the first row is the scaling chosen for the main analysis. The fourth row represents the final chose projection using estimated cases per the state space model. Columns 1 and 2 display countries' position on the canonical path between 1993 and 2017 (thin lines) by region (color), with non-weighted and population weighted region averages overlaid (column 1 and 2, respectively). Columns 3 and 4 display countries' speed of movement along the canonical path between 1993 and 2017 (thin lines) by region (color), with non-weighted and population weighted region averages overlaid (column 1 and 2, respectively).

References and Notes

1. Centers for Disease Control and Prevention, Global Health: Measles, Rubella, & Congenital Rubella Syndrome (CRS) Elimination; www.cdc.gov/globalhealth/measles/elimination.htm.

2. Pan American Health Organization, Epidemiological Update: Measles. 24 October 2018; www.paho.org/hq/index.php?option=com_docman&view=download&category_slug=measles-2204&alias=46783-24-october-2018-measles-epidemiological-update&Itemid=270&lang=en.

3. World Health Organization, A Guide to Introducing a Second Dose of Measles Vaccine into Routine Immunization Schedules; apps.who.int/iris/bitstream/10665/85900/1/WHO_IVB_13.03_eng.pdf.

4. World Health Organization, Planning and Implementing High-Quality Supplementary Immunization Activities for Injectable Vaccines Using an Example of Measles and Rubella Vaccines: Field Guide (2016); https://www.who.int/immunization/diseases/measles/SIA-Field-Guide.pdf.

5. O. N. Bjørnstad, B. F. Finkenstädt, B. T. Grenfell, Dynamics of measles epidemics: Estimating scaling of transmission rates using a time series SIR model. *Ecol. Monogr.* **72**, 169–184 (2002). doi:10.2307/3100023

6. B. T. Grenfell, O. N. Bjørnstad, J. Kappey, Travelling waves and spatial hierarchies in measles epidemics. *Nature* **414**, 716–723 (2001). doi:10.1038/414716a Medline

7. M. J. Ferrari, R. F. Grais, N. Bharti, A. J. K. Conlan, O. N. Bjørnstad, L. J. Wolfson, P. J. Guerin, A. Djibo, B. T. Grenfell, The dynamics of measles in sub-Saharan Africa. *Nature* **451**, 679–684 (2008). doi:10.1038/nature06509 Medline

8. N. Bharti, A. Djibo, M. J. Ferrari, R. F. Grais, A. J. Tatem, C. A. McCabe, O. N. Bjornstad, B. T. Grenfell, Measles hotspots and epidemiological connectivity. *Epidemiol. Infect.* **138**, 1308–1316 (2010). doi:10.1017/S0950268809991385 Medline

9. B. Finkenstädt, B. Grenfell, Empirical determinants of measles metapopulation dynamics in England and Wales. *Proc. Biol. Sci.* **265**, 211–220 (1998). doi:10.1098/rspb.1998.0284 Medline

10. Materials and methods are available as supplementary materials.

11. See web application developed with R package Shiny, to view all countries' positions on the canonical path between 1980 and 2017 (http://iddynamics.jhsph.edu/apps/shiny/measlescanonicalpath/).

12. S. Chen, J. Fricks, M.J. Ferrari, Tracking measles infection through non-linear state space models. *J. R. Stat. Soc. Ser. C Appl. Stat.* **61**, 117–134 (2011). doi:10.1111/j.1467-9876.2011.01001.x

13. E. Simons, M. Ferrari, J. Fricks, K. Wannemuehler, A. Anand, A. Burton, P. Strebel, Assessment of the 2010 global measles mortality reduction goal: Results from a model of surveillance data. *Lancet* **379**, 2173–2178 (2012). doi:10.1016/S0140-6736(12)60522-4 Medline

14. B. T. Grenfell, O. N. Bjornstad, B. F. Finkenstadt, Dynamics of Measles Epidemics: Scaling Noise, Determinism, and Predictability with the TSIR Model. *Ecol. Monogr.* **72**, 185–202 (2002). doi:10.1890/0012-9615(2002)072[0185:DOMESN]2.0.CO;2

15. M. S. Bartlett, Measles Periodicity and Community Size. *J. R. Stat. Soc. Ser. A* **120**, 48 (1957). doi:10.2307/2342553

16. M. S. Bartlett, The Critical Community Size for Measles in the United States. *J. R. Stat. Soc. Ser. A* **123**, 37 (1960). doi:10.2307/2343186

17. F. L. Black, Measles endemicity in insular populations: Critical community size and its evolutionary implication. *J. Theor. Biol.* **11**, 207–211 (1966). doi:10.1016/0022-5193(66)90161-5 Medline

18. D. A. Griffiths, The Effect of Measles Vaccination on the Incidence of Measles in the Community. *J. R. Stat. Soc. Ser. A* **136**, 441 (1973). doi:10.2307/2344999

19. T. P. Van Boeckel, S. Takahashi, Q. Liao, W. Xing, S. Lai, V. Hsiao, F. Liu, Y. Zheng, Z. Chang, C. Yuan, C. J. E. Metcalf, H. Yu, B. T. Grenfell, Hand, Foot, and Mouth Disease in China: Critical Community Size and Spatial Vaccination Strategies. *Sci. Rep.* **6**, 25248 (2016). doi:10.1038/srep25248 Medline

20. S. Takahashi, C. J. E. Metcalf, M. J. Ferrari, W. J. Moss, S. A. Truelove, A. J. Tatem, B. T. Grenfell, J. Lessler, Reduced vaccination and the risk of measles and other childhood infections post-Ebola. *Science* **347**, 1240–1242 (2015). doi:10.1126/science.aaa3438 Medline

21. M. Keeling, B. Grenfell, Stochastic dynamics and a power law for measles variability. *Philos. Trans. R. Soc. Lond. B Biol. Sci.* **354**, 769–776 (1999). doi:10.1098/rstb.1999.0429 Medline

22. H. Rosling, A. R. Rönnlund, O. Rosling, *Factfulness: Ten Reasons We're Wrong About the World–and Why Things Are Better than You Think* (Flatiron Books, 2018).

23. World Bank, Population, total; http://data.worldbank.org/indicator/SP.POP.TOTL.

24. World Bank, Birth rate, crude (per 1,000 people); http://data.worldbank.org/indicator/SP.DYN.CBRT.IN.

25. World Health Organization, Measles, 1st dose (MCV): immunization coverage estimates by country; http://apps.who.int/gho/data/node.main.A826.

26. World Health Organization, Measles: Reported cases by country; http://apps.who.int/gho/data/view.main.1540_62?lang=en.

27. C. J. E. Metcalf, J. Lessler, P. Klepac, F. Cutts, B. T. Grenfell, Impact of birth rate, seasonality and transmission rate on minimum levels of coverage needed for rubella vaccination. *Epidemiol. Infect.* **140**, 2290–2301 (2012). doi:10.1017/S0950268812000131 Medline

28. C. J. E. Metcalf, J. Lessler, P. Klepac, A. Morice, B. T. Grenfell, O. N. Bjørnstad, Structured models of infectious disease: Inference with discrete data. *Theor. Popul. Biol.* **82**, 275–282 (2012). doi:10.1016/j.tpb.2011.12.001 Medline

29. Population Division, Department of Economic, Social Affairs and United Nations. wpp2015:

World Population Prospects 2015. R package version 1.1-0. https://CRAN.R-project.org/package=wpp2015 (2016).

30. V. M. Cáceres, P. M. Strebel, R. W. Sutter, Factors determining prevalence of maternal antibody to measles virus throughout infancy: A review. *Clin. Infect. Dis.* **31**, 110–119 (2000). doi:10.1086/313926 Medline

31. H. A. Gans, A. M. Arvin, J. Galinus, L. Logan, R. DeHovitz, Y. Maldonado, Deficiency of the humoral immune response to measles vaccine in infants immunized at age 6 months. *JAMA* **280**, 527–532 (1998). doi:10.1001/jama.280.6.527 Medline

32. J. Mossong, N. Hens, M. Jit, P. Beutels, K. Auranen, R. Mikolajczyk, M. Massari, S. Salmaso, G. S. Tomba, J. Wallinga, J. Heijne, M. Sadkowska-Todys, M. Rosinska, W. J. Edmunds, Social contacts and mixing patterns relevant to the spread of infectious diseases. *PLOS Med.* **5**, e74 (2008). doi:10.1371/journal.pmed.0050074 Medline

33. T. Hastie, R. Tibshirani, Generalized Additive Models. *Stat. Sci.* **1**, 297–310 (1986). doi:10.1214/ss/1177013604

34. M. Graham, J. Lessler, A. Azman, A. K. Winter, HopkinsIDD/MeaslesCanonicalPath: Canonical path code at time of March 2019 submission, Zenodo (2019); https://doi.org/10.5281/zenodo.2586459
